# Bioactive plant waste components targeting oral bacterial pathogens as a promising strategy for biofilm eradication

**DOI:** 10.3389/fchem.2024.1406869

**Published:** 2024-08-09

**Authors:** Saima Mashal, Aisha Siddiqua, Niamat Ullah, Rabia Baloch, Momin Khan, Syed Zia Ul Hasnain, Muhammad Imran Aziz, Elchin Huseynov, Dragica Selakovic, Gvozden Rosic, Trobjon Makhkamov, Akramjon Yuldashev, Sokhib Islamov, Nilufar Abdullayeva, Uktam Khujanazarov, Adnan Amin

**Affiliations:** ^1^ Gomal Center of Biochemistry and Biotechnology (GCBB), Gomal University, Dera Ismail Khan, Khyber Pakhtunkhwa, Pakistan; ^2^ Natural Products Research Lab, Department of Pharmacognosy, Faculty of Pharmacy, Gomal University, Dera Ismail Khan, Khyber Pakhtunkhwa, Pakistan; ^3^ Allama Iqbal Teaching Hospital, Dera Ghazi Khan, Pakistan; ^4^ Department of Microbiology, Institute of Pathology and Diagnostic Medicine, Khyber Medical University, Peshawar, Khyber Pakhtunkhwa, Pakistan; ^5^ Department of Pharmacognosy, Bahauddin Zakariya University, Multan, Pakistan; ^6^ Azerbaijan Medical University, Baku, Azerbaijan; ^7^ Department of Physiology, Faculty of Medical Sciences, University of Kragujevac, Kragujevac, Serbia; ^8^ Department of Forestry and Land Scape Design, Tashkent State Agrarian University, Tashkent, Uzbekistan; ^9^ Department of Ecology and Botany Andijan State University, Andijan, Uzbekistan; ^10^ Department of Technology of Storage and Processing of Agricultural Products, Tashkent State Agrarian University, Tashkent, Uzbekistan; ^11^ Department of Biology Teaching Methodology, Jizzakh State Pedagogical University, Jizzakh, Uzbekistan; ^12^ Department of Botany and Ecology, Tashkent State Pedagogical University, Tashkent, Uzbekistan

**Keywords:** oral pathogens, bioremediation, polyphenolic compounds, docking, *Paenibacillus dendritiformis*

## Abstract

The significance of this study lies in its exploration of bioactive plant extracts as a promising avenue for combating oral bacterial pathogens, offering a novel strategy for biofilm eradication that could potentially revolutionize oral health treatments. Oral bacterial infections are common in diabetic patients; however, due to the development of resistance, treatment options are limited. Considering the excellent antimicrobial properties of phenolic compounds, we investigated them against isolated oral pathogens using *in silico* and *in vitro* models. We performed antibiogram studies and minimum inhibitory concentration (MIC), antibiofilm, and antiquorum sensing activities covering phenolic compounds. Bacterial strains were isolated from female diabetic patients and identified by using 16S rRNA sequencing as *Pseudomonas aeruginosa*, *Bacillus chungangensis*, *Bacillus paramycoides*, and *Paenibacillus dendritiformis*. Antibiogram studies confirmed that all strains were resistant to most tested antibiotics except imipenem and ciprofloxacin. Molecular docking analysis revealed the significant interaction of rutin, quercetin, gallic acid, and catechin with transcription regulator genes *1RO5*, *4B2O*, and *5OE3*. All tested molecules followed drug-likeness rules except rutin. The MIC values of the tested compounds varied from 0.0625 to 0.5 mg/mL against clinical isolates. Significant antibiofilm activity was recorded in the case of catechin (73.5% ± 1.6% inhibition against *B. paramycoides*), cinnamic acid (80.9% ± 1.1% inhibition against *P. aeruginosa*), and vanillic acid and quercetin (65.5% ± 1.7% and 87.4% ± 1.4% inhibition, respectively, against *B. chungangensis*) at 0.25–0.125 mg/mL. None of the phenolic compounds presented antiquorum sensing activity. It was, therefore, concluded that polyphenolic compounds may have the potential to be used against oral bacterial biofilms, and further detailed mechanistic investigations should be performed.

## 1 Introduction

The natural flora of the oral cavity is very diverse, with over 500 distinct bacterial species from several phyla reported (Aas et al., 2005). These bacteria occur in a homeostatic mode, and a slight imbalance can accelerate the development of bacterial infections in the oral cavity (Minhas et al., 2019). Tooth erosion (due to acid production by bacteria), periodontitis, dental abscess, cavities, and gingivitis are common oral infections (Loesche, 1996). Oral cancer is the worst case of longer and persistent bacterial infections of the oral cavity (Siqueira and Rôças, 2022). Most oral infections occur due to the bacterial capability to adhere within small gingival openings and outer tooth surfaces, leading to the development of biofilms (Hora and Patil, 2022).

The bacterial biofilm is a layer of adherent bacteria on oral cavity surfaces covered by a capsular matrix (Verderosa et al., 2019; Schulze et al., 2021). Biofilms generally comprise multispecies of bacteria that demonstrate quite diverse and versatile microbial interactions (Zhao et al., 2023). The biofilms mainly produce antimicrobial resistance due to horizontal gene transfer, oxygen gradient, persister cells, efflux pumps, and eDNA (Rather et al., 2021). To overcome this serious health concern, antimicrobial agents and/or their combination are used in clinical settings to eradicate bacterial biofilms; however, the side effects are the main concerns (Cunha, 2001).

Since ancient times, herbs including *Syzygium aromaticum*, *Salvadora persica*, and *Juglans regia* have been used in traditional remedies for the treatment of toothache and bacterial infections (Zakavi et al., 2013; Megersa et al., 2019; Mekhemar et al., 2021). Researchers have been working exclusively on plant-based compounds as alternative drug candidates for oral bacterial infections (Nagpal and Sood, 2013) since they are safe, biocompatible, cost-effective, and non-resistant compared to their synthetic counterparts (Kumar et al., 2013). Plant polyphenolic compounds are a diverse class of compounds (above 8,000), including flavonoids, phenolic acids, lignans, and tannins (Cheynier, 2005). These are characterized by the presence of more than two phenolic groups attached to two phenyl rings (Hanhineva et al., 2010). Polyphenolic compounds are associated with strong antioxidant potential and possess high penetration capabilities that result in significant damage to the cell membrane (Zamuz et al., 2021). Furthermore, polyphenolic compounds possess significant antimicrobial properties due to the inhibition of several virulence factors, disruption of the lipid membrane, and strong antibiofilm features (Rana et al., 2022). Considering diverse biological activities related to polyphenolic compounds and the emergence of resistance toward oral bacteria, the current study aimed to analyze polyphenolic compounds against resistant clinical isolates from the oral cavity using *in vitro* and *in vivo* models.

## 2 Materials and methods

### 2.1 Chemicals and growth media

All chemicals and growth media used in the investigation were of analytical grade. Chemicals including ferulic acid, gentisic acid, gallic acid, rutin, quercetin, catechin, caffeic acid, syringic acid, vanillic acid, and Congo red were purchased from Sigma-Aldrich (St. Louis, MO, United States). The growth media, including Luria Bertani (LB) agar, nutrient agar, and triple sugar iron agar (TSIA), were obtained from HiMedia (Mumbai, India), whereas MacConkey agar and eosin methylene blue (EMB) agar were purchased from Oxoid (Hampshire, United Kingdom). The standard strains, including *Escherichia coli* (ATCC 25922), *Klebsiella pneumoniae* (ATCC BAA-1705), *Pseudomonas aeruginosa* (ATCC 15442), and *Staphylococcus aureus* (ATCC 33862), were obtained from Microbiologics™ (United Kingdom). The biomarker strain *Chromobacterium violaceum* was obtained from DSMZ, Germany (DSM 30191).

### 2.2 Sample processing

Dental plaques from female diabetic patients were obtained by a dentist at DHQ Teaching Hospital D.I. Khan, Pakistan, with informed patient consent. The ethical approval for the investigation was obtained (Approval No. 331/ERB/GU/2022). The dental plaques were shifted to growth media (nutrient agar) and processed further in the laboratory. After 24 h of incubation, the bacterial cultures were spread on differential media, including MacConkey agar, TSIA, and EMB agar, for preliminary bacterial purification and identification.

### 2.3 Preliminary detection of biofilm formation

Purified bacterial strains were analyzed on Congo red agar by a standard procedure (Vestby et al., 2020). In brief, Congo red (40 μg/mL) and Coomassie brilliant blue (40 μg/mL) were added to LB agar without NaCl. The tested bacteria were inoculated onto each plate and incubated at 37°C for 24–72 h. The colony morphology was examined at each time interval, and the development of black–brown colonies (pellicle formation) was considered biofilm producer strains.

### 2.4 Bacterial identification through 16S rRNA sequencing

For the authentic identification of bacteria, 16S rRNA sequencing was used with several steps, including DNA extraction, DNA quantification, 16S rRNA gene qPCR, and 16S rRNA gene sequencing (detailed protocols are mentioned in Supplementary Material).

### 2.5 *In silico* analysis

#### 2.5.1 Drug likeness, PASS analysis, and ADMET analysis

The canonical Simplified Molecular-Input Line-Entry System (SMILES) of the tested compounds was obtained from PubChem. Drug likeness and ADMET analysis of polyphenolic compounds was performed using several *in silico* tools, including SwissADME, pkCSM, and the Molinspiration tool (Amin et al., 2020). The canonical SMILES of the compounds was obtained from PubChem and loaded on the INPUT area of software. The generated structure was confirmed, and finally, analyses were performed.

### 2.6 Molecular docking

The transcription regulator genes (*2V50*, *50E3*, and *2XCT*) were selected based on information from the literature (Aparna et al., 2014; Patel and Patel, 2014; Shaker et al., 2020). The X-ray crystallographic structures were obtained from the Protein Data Bank (PDB). The 3D structures of the tested compounds were obtained from PubChem in SDF format, and active site prediction was accomplished using the online tool CASTp 3.0. Molecular docking was accomplished using the Lamarckian genetic algorithm included in AutoDock v4.2.6. Docking interaction analysis was performed using LigPlot^+^, and Accelrys DS Visualizer 2.0 and PyMOL were used to analyze the best-docked molecules with high free energy [ΔG]. For each regulator gene, nine poses were generated, and all were classified based on their RMSD values.

### 2.7 Biological assays

#### 2.7.1 Antibiogram development

Antibiograms (antimicrobial resistance pattern) were determined using a disk diffusion method (Myemba et al., 2022). In brief, antimicrobial susceptibility testing disks were placed aseptically in pre-bacterial-loaded culture medium (nutrient agar) plates and incubated for 24 h at 37°C. Afterward, the growth of bacteria around each susceptibility disk was measured, and the results were recorded.

#### 2.7.2 Determination of the minimum inhibitory concentration

The antimicrobial activity of polyphenolic compounds was determined using a standard method (Rafey et al., 2021). In brief, 50 μL of the tested compound (0.125–0.25 mg/mL) was loaded into each well of 96-well microplates and mixed with 50 μL of bacterial culture (previously adjusted with 0.5 McFarland standard). The plates were then incubated for 24 h at 37°C. Afterward, a Resazurin solution (0.015%, 40 μL) was added to each well and incubated further for 2 h. The results were recorded using a colorimetric method.

#### 2.7.3 Antibiofilm assay

The antibiofilm activity of the tested samples was determined using a modified method (Rafey et al., 2021). In brief, bacterial cultures (225 μL, adjusted with 0.5 McFarland standard) were loaded into each well of 96-well microplates and mixed with the tested compounds (25 μL, 0.125–0.25 mg/mL) and incubated at 37 °C for 24–48 h. To quantify biofilm formation, the crystal violet staining method was used. After staining, the biofilms were washed thrice with sterilized saline and mixed with an acetic acid solution to dissolve the biofilms. Finally, absorbance at 592 nm was recorded using a 96-well microplate reader. The % inhibition was calculated using the following formula:

$$\%\text{ inhibition}=\left[1\;-\;\left(\text{absorbance of the sample}/\text{absorbance of the control}\right)\right]\times 100.$$

#### 2.7.4 Antiquorum sensing

Antiquorum sensing activity of the selected phenolic compounds was determined using a standard technique with slight modifications (Rafey et al., 2021). The sterilized culture medium plates (LB agar plates) were prepared, and a fresh strain (24 h) of *C. violaceum* was inoculated on each plate. A 6-mm blank disk loaded with the tested compound (0.125–0.25 mg/mL) was placed on each plate and incubated at 30°C for 24–48 h. Afterward, the zones of inhibition around each disk were measured, and the results were recorded.

## 3 Results

### 3.1 Bacterial identification and biofilm formation assays

Bacterial identification through 16S rRNA sequencing confirmed bacterial strains like *Bacillus chungangensis*-1, *P. aeruginosa*, *Bacillus paramycoides*, *Bacillus chungangensis-2*, and *Paenibacillus dendritiformis*. The identified strains were processed for biofilm formation, and it was observed that all isolated clinical strains were biofilm-positive on Congo red agar (Table 1; Supplementary Material).

**TABLE 1:** Bacterial identification by 16S rRNA sequencing results.

	Strain blast	Length	
		Normal	Q20^a^
1	*Pseudomonas aeruginosa*	1,208	958
2	*Bacillus chungangensis*	945	687
3	*Bacillus paramycoides*	1,149	1,059
4	*Bacillus chungangensis*	1,194	1,107
5	*Paenibacillus dendritiformis*	1,277	1,118

^a^ Quality score greater than 20 (sequencing error rate less than 1%, purity 99%).

### 3.2 *In silico* analysis

#### 3.2.1 Drug likeness, bioavailability, and ADMET analysis

**FIGURE 1:**
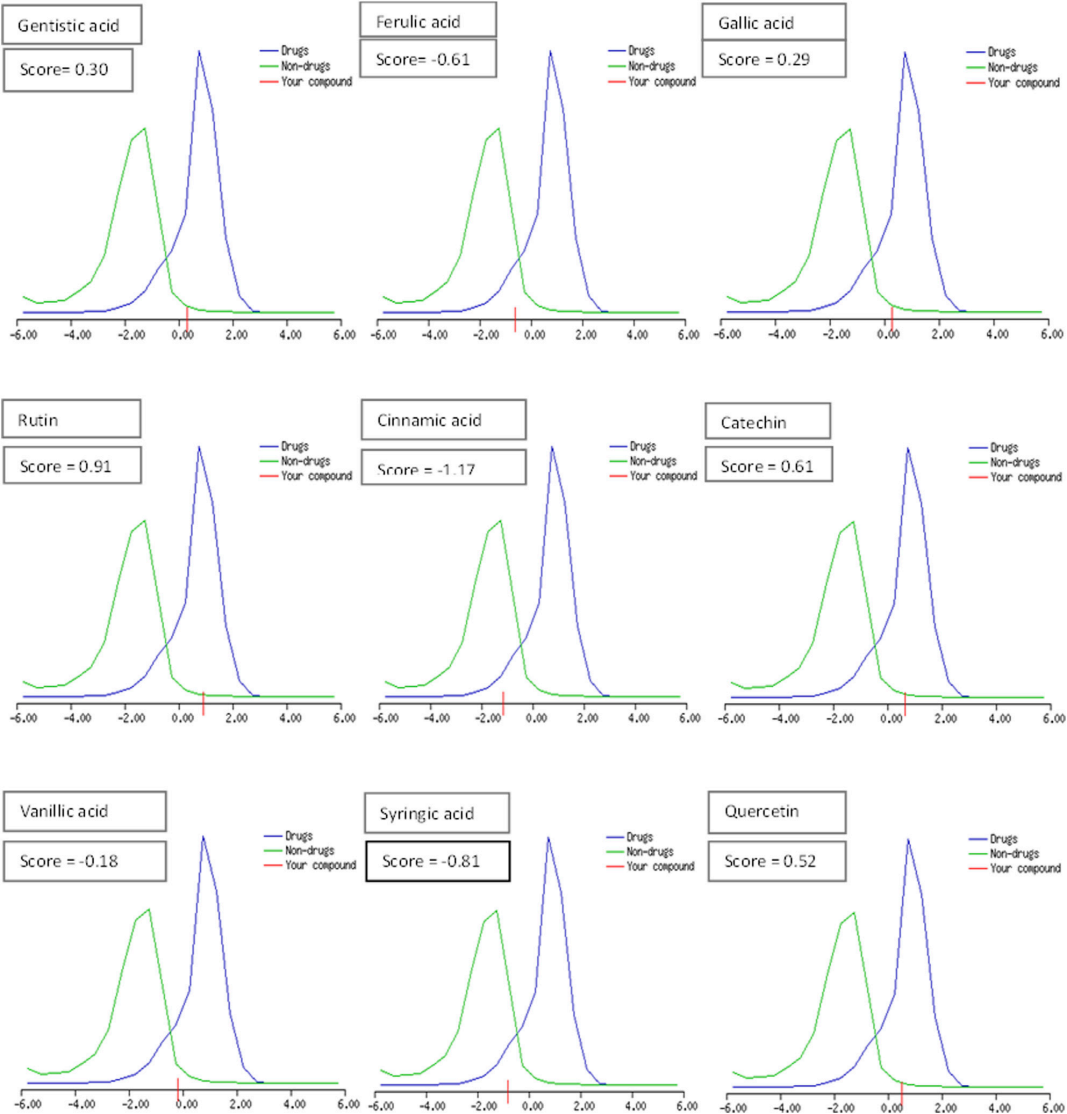
Drug-likeness score of the phenolic compounds (the blue peak shows standard drug properties [+1 to −1], the green peak shows non-drug properties [lower than 1], and red shows our tested molecule).

**FIGURE 2:**
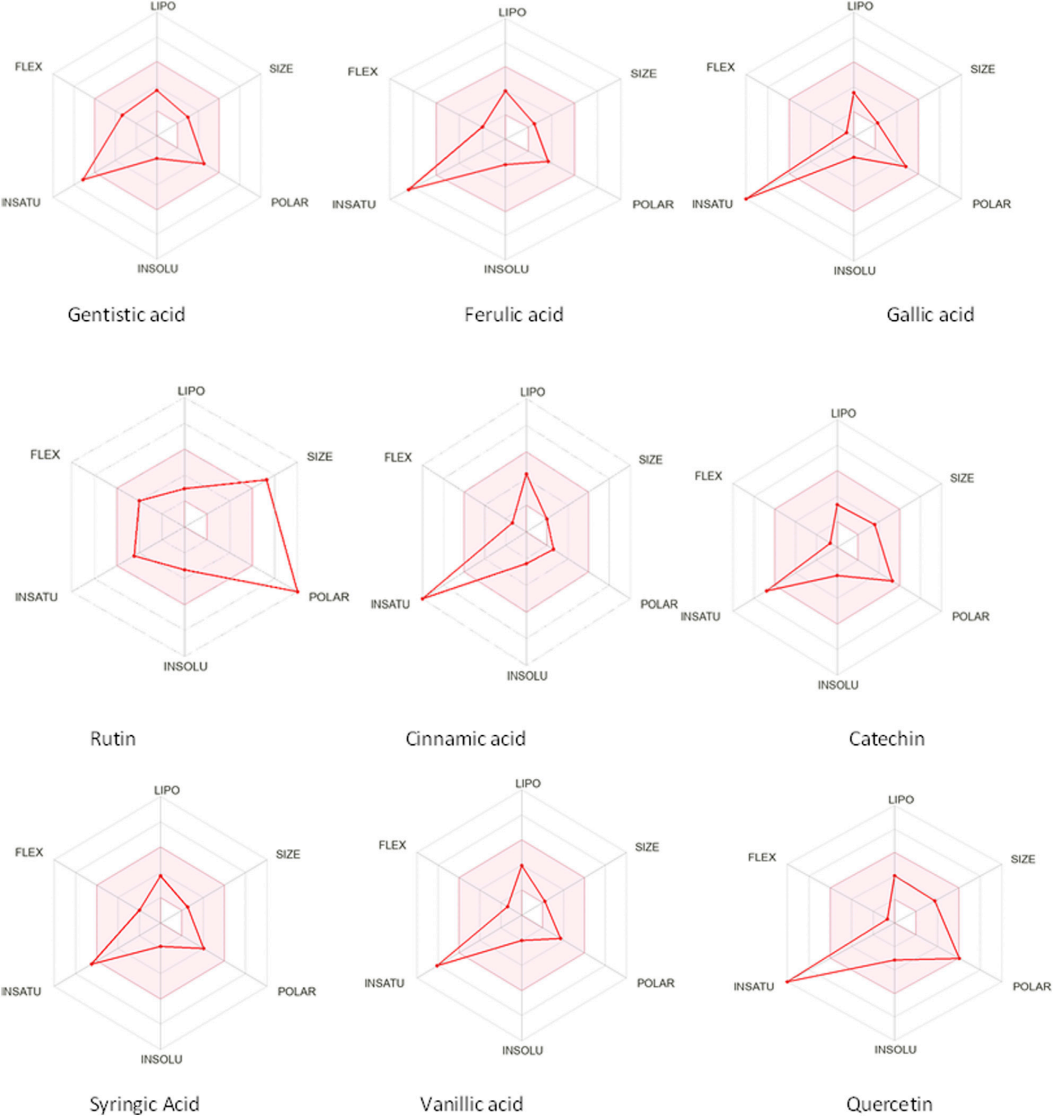
Bioavailability radars of the phenolic compounds (the pink area shows the optimal region, whereas the white area is an indication of non-optimal values that can effect bioavailability).

**FIGURE 3:**
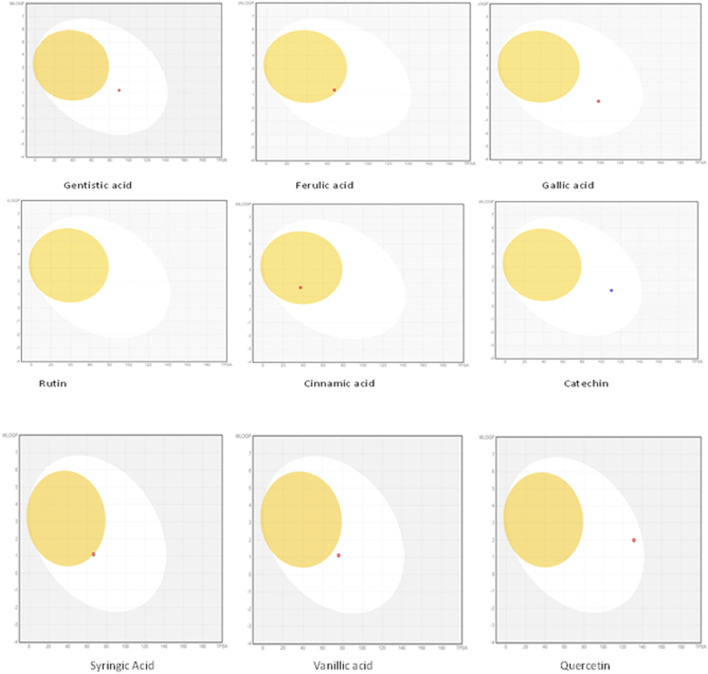
Boiled egg models of the phenolic compounds (the yellow area shows that the drug can cross the blood–brain barrier, whereas the white area is an indication of permeability through the gastrointestinal tract).

The drug likeness was determined by the Lipinski rule of five using the Molinspiration tool. It was noted that all tested polyphenolic compounds exhibited compliance with the Lipinski rule except rutin, which showed four violations (Table 2; Figure 1). Bioavailability profiling of compounds was analyzed by using a bioavailability radar (Figure 2). It was evident that all tested molecules were within the colored area, indicating drug-like features except INSATU (unsaturation). A boiled egg model was used for the determination of compound bioavailability in the brain (ability to cross the blood–brain barrier). Our findings indicate that cinnamic acid, ferulic acid, and vanillic acid are capable of permeating the brain, whereas all other tested molecules are only absorbed from the gastrointestinal tract (Figure 3).

**TABLE 2:** Lipinski properties of the tested polyphonic compounds.

S. no	Compound	Molecular weight < 500 Da	Log *p* < 5	H-bond donor (5)	H-bond accepter < 10	Number of violations
1	Gentisic acid	238.195	1.2354	1	5	0
2	Ferulic acid	194.186	1.4986	2	3	0
3	Gallic acid	170.02	0.78	4	2	0
4	Rutin	610.521	−1.6871	10	16	4
5	Cinnamic acid	148.05	2.22	1	2	0
6	Catechin	290.08	0.53	5	6	0
7	Quercetin	302.04	1.19	5	7	0
8	Caffeic acid	180.04	1.27	3	4	0
9	Syringic acid	198.174	1.1076	2	5	0
10	Vanillic acid	168.04	1.20	2	4	0

ADMET profiling was performed using Molsoft and SwissADME tools. It was noted that flavonoids, including rutin, quercetin, and catechin, presented high oral absorption, whereas gallic acid and rutin showed poor intestinal absorption. All other tested molecules presented >70% absorption rates (Tables 3, 4). Similarly, low skin permeability was noted in all tested molecules. Furthermore, a low volume of distribution (Vd) was recorded in the case of the tested compounds. Furthermore, our findings showed that most of the tested molecules were neither substrates nor inhibitors of cytochrome P450 (Tables 3, 4). Finally, clearance of rutin, quercetin, and catechin was predicted from the renal route, and no or little toxicity was recorded in the case of all tested molecules (Tables 3, 4).

**TABLE 3:** ADMET properties of the phenolic compounds (A).

Properties	Tested molecules				
	Gentisic acid	Ferulic acid	Cinnamic acid	Rutin	Quercetin
TPSA (A°)	89.80 Å^2^	66.76 Å^2^	37.30 Å^2^	269.43 Å^2^	131.36 Å^2^
Consensus log Po/w	1.28	1.36	1.79	−1.51	1.23
Absorption					
Water solubility (log mol/L)	−1.784	−1.603	−2.08	−3.327	−3.275
CaCo2 permeability (log Papp in 10^−6^ cm/s)	0.34	0.279	1.449	−0.791	0.076
Intestinal absorption (human) (% absorbed)	82.884	93.921	96.809	28.495	73.104
Skin permeability (log Kp)	−2.964	−2.881	−2.538	−2.737	−3.368
P-Glycoprotein substrate	Yes	Yes	Yes	Yes	Yes
P-Glycoprotein I inhibitor	No	No	No	Yes	No
P-Glycoprotein II inhibitor	No	No	No	Yes	No
Distribution					
VDss (human, log L/kg)	−1.229	−0.851	−0.565	−1.597	−1.133
Fraction unbound (human) (Fu)	0.452	0.431	0.395	0.419	0.275
BBB permeability (logBB)	−0.592	−0.242	0.256	−2.215	−1.065
CNS permeability (log PS)	−3.224	−2.573	−1.443	−4.842	−3.071
Metabolism					
CYP2D6 substrate	No	No	No	No	No
CYP3A4 substrate	No	No	No	Yes	No
CYP1A2 inhibitor	No	No	Yes	No	Yes
CYP2C19 inhibitor	No	No	No	No	No
CYP2C9 inhibitor	No	No	No	No	No
CYP2D6 inhibitor	No	No	No	No	No
CYP3A4 inhibitor	No	No	No	No	No
Excretion					
Total clearance (logml/min/kg)	0.838	0.621	0.797	0.187	0.488
Renal OCT2 substrate	No	No	No	No	No
Toxicity					
AMES toxicity	Yes	No	No	No	Yes
hERG I inhibitor	No	No	No	No	No
hERG II inhibitor	No	No	No	Yes	No
Hepatotoxicity	No	No	No	No	No
Skin sensitization	No	No	Yes	No	No

**TABLE 4:** ADMET properties of the phenolic compounds (B).

Property	Tested molecules			
	Gallic acid	Catechin	Vanillic acid	Syringic acid
TPSA (A°)	97.99 Å^2^	110.38 Å^2^	66.76 Å^2^	75.99 Å^2^
Consensus log Po/w	0.21	0.83	1.08	0.99
Absorption				
Water solubility (logmol/L)	−0.723	−2.808	−0.992	−2.223
CaCo2 permeability (log Papp in 10–6 cm/s)	−0.467	−0.38	0.199	0.495
Intestinal absorption (human) (% absorbed)	50.311	71.562	75.448	73.076
Skin permeability (log Kp)	−3.084	−3.603	−2.941	−2.735
P-Glycoprotein substrate	Yes	Yes	Yes	Yes
P-Glycoprotein I inhibitor	No	No	No	No
P-Glycoprotein II inhibitor	No	No	No	No
Distribution				
VDss (human, log L/kg)	−1.078	−0.79	−0.907	−1.443
Fraction unbound (human) (Fu)	0.565	0.326	0.496	0.601
BBB permeability (logBB)	−0.93	−0.905	−0.295	−0.191
CNS permeability (log PS)	−2.816	−3.146	−2.601	−2.701
Metabolism				
CYP2D6 substrate	No	No	No	No
CYP3A4 substrate	No	No	No	No
CYP1A2 inhibitor	No	No	No	No
CYP2C19 inhibitor	No	No	No	No
CYP2C9 inhibitor	No	No	No	No
CYP2D6 inhibitor	No	No	No	No
CYP3A4 inhibitor	No	No	No	No
Excretion				
Total clearance (logml/min/kg)	0.55	0.215	0.626	0.646
Renal OCT2 substrate	No	No	No	No
Toxicity				
AMES toxicity	No	Yes	No	No
hERG I inhibitor	No	No	No	No
hERG II inhibitor	No	No	No	No
Hepatotoxicity	No	No	No	No
Skin sensitization	No	No	No	No

### 3.3 Molecular docking

**FIGURE 4:**
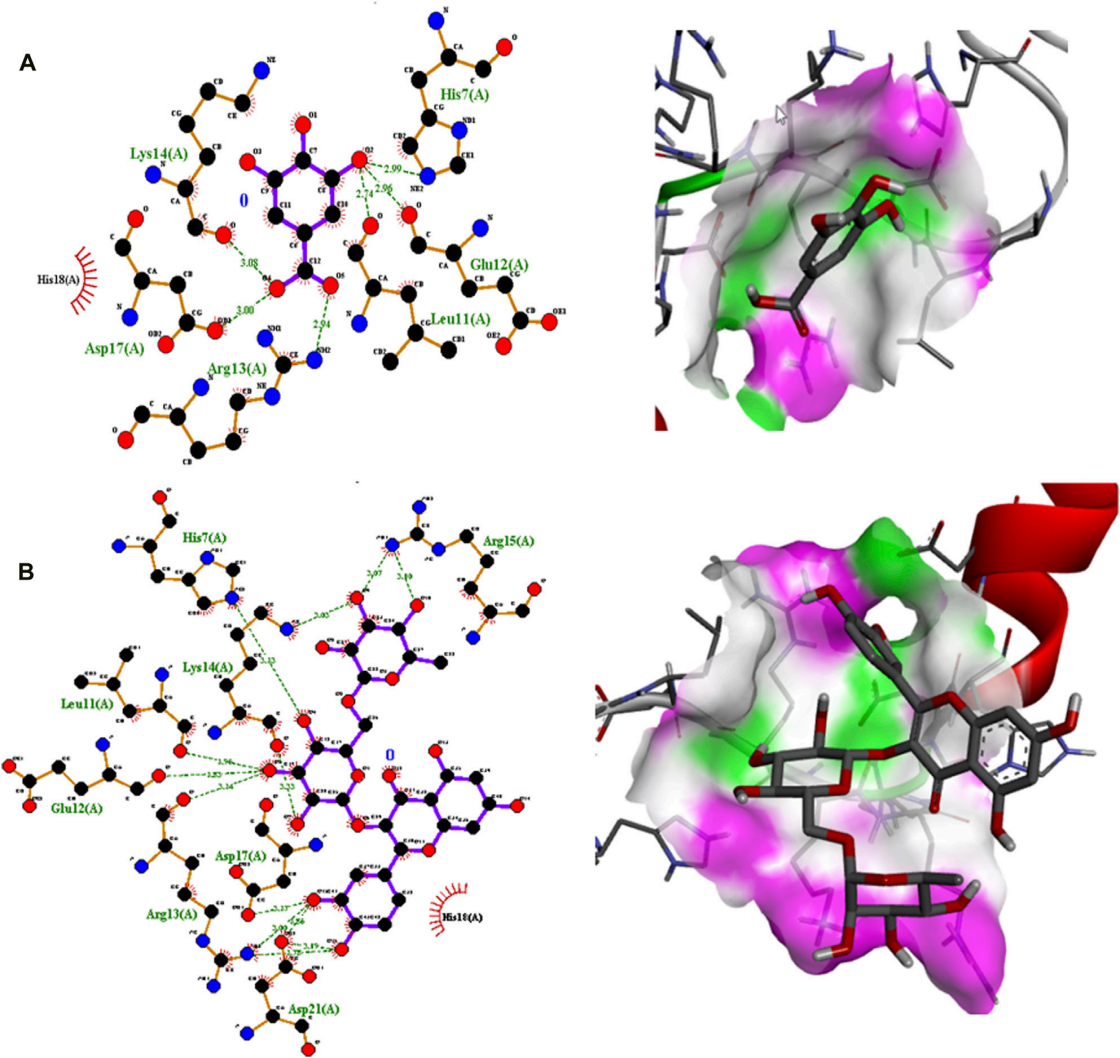
Interaction analysis of gallic acid pose 1 **(A)** and rutin pose 1 **(B)** with transcription regulator IRO5.

**FIGURE 5:**
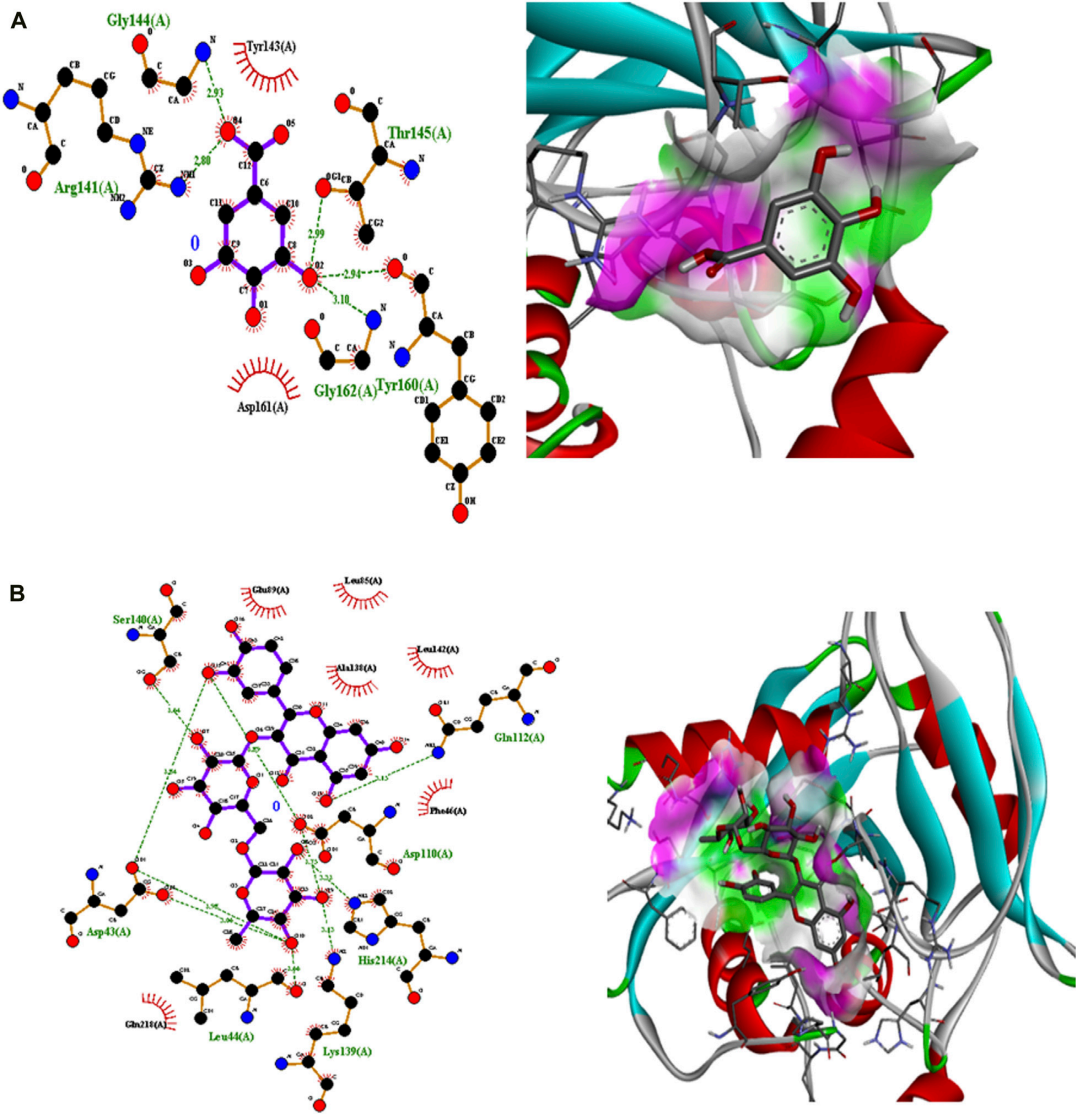
Interaction analysis of gallic acid pose 8 **(A)** and rutin pose 1 **(B)** with transcription regulator 4B2O.

Polyphenolic compounds were docked with regulator genes for biofilm production and quorum sensing, including *1ROS*, *4B2O*, and *5OE3*. Docking with *1ROS* showed a strong interaction (−5.8 ΔG [kJ mol^‒1^]). Amino acids including Asp21, Arg13, Asp17, Glu12, Leu11, Lys14, His7, and Arg15 at pose 1 participated through H-bonding interactions. The interaction analysis of all phenolic compounds is presented in Table 5; Figure 4. Docking of polyphenolic compounds with the *Bacillus* species biofilm target gene (*4B2O*) showed the highest H-bonding interactions with rutin. In this case, seven amino acid interactions were recorded, namely, Leu44, Asp43, Ser140, Gln112, Asp110, His21, and Lys139, through high free binding energy [−8.1 ΔG (kJ mol^‒1^)]. The interaction analysis of all phenolic compounds with *4B2O* is shown in Table 5; Figure 5. Lastly, compounds were docked against the biofilm producer gene *PqsA* (PDB ID = 5OE3). In the case of rutin, a significant H-bonding interaction was observed with high free energy [−9.4 ΔG (kJ mol‒1)]. Six amino acids, namely, Gly300, Thr304, Glu305, Asp382, His394, and Gly279, at pose 1 showed a strong H-bonding interaction with the target site, whereas amino acid residues including Ala278, Gly302, Ile301, Ala303, Tyr378, Thr164, and Ser28 showed van der Waals interactions, π–sigma, π–π, and π–alkyl interactions (Table 5; Figure 4). In the case of quercetin, a strong H-bonding interaction was recorded with amino acid residues, including Gly279, Gly300, Thr304, Glu305, Asp382, and His394 at pose rank 1. The interaction was quite stable with high fee binding energy [−8.4 ΔG (kJ mol‒1)]. The other amino acids in the active pocket, including Pro281, Ala278, Gly302, Ile301, Ala303, Tyr378, Thr164, and Ser280, interacted with quercetin through hydrophobic interactions (Table 5; Figure 4). Both H-bonding and hydrophobic interactions are of great significance since they determine the stability and structure of proteins that are crucial in therapeutic effects. Molecular docking investigations revealed a strong interaction of polyphenolic compounds, especially flavonoids, with target sites, which may indicate the potential inhibitory effect of these compounds on bacterial growth.

**TABLE 5:** Docking score and H and non-H-bond interactions of tested compounds (A).

Compound	Binding free energy ΔG (kJ mol^‒1^)	Pose number	Number of H bonds	Amino acid interaction residues	Hydrophobic interactions with amino acids
1RO5					
Caffeic acid	−5.2	1	2	Asp17, Asp21, Glu12, Arg13, and His7	His18, Lys14, and Leu11
Catechin	−5.3	3	3	Asp21, Arg13, Leu11, and Glu12	Lys14, His7, and His18
Cinnamic acid	−4.0	5	3	His7, Glu12, and Asn9	Lys4, Ala6, Arg5, and Ala2
Ferulic acid	−4.6	1	3	Gln42, His24, and Asn 41	Ser39, Lys37, Arg40, Lys20, Phe23, and Arg27
Gallic acid	−4.7	2	6	Arg13, Asp17, Lys14, His7, Glu12, and Leu11	His18
Gentistic acid	−4.3	2	4	Arg27, Val30, Gln34, and Pro31	Leu33 and Asp28
Quercetin	−7.5	1	5	Thr144, Phe105, Ile107, Arg30, and Thr145	Phe27, Trp33, Phe177, Ser109, Val126, and Val148
Rutin	−5.8	1	8	Asp21, Arg13, Asp17 Glu12, Leu11, Lys14, His7, and Arg15	His18
Syringic acid	−4.5	1	2	Asp17 and Arg13	His7, Lys14, Arg15, His18, and Leu11
Vanillic acid	−4.2	1	3	His7, Lys4, and Arg5	Asn9, Asp3, Ala2, and Glu12
4B2O					
Caffeic acid	−4.5	5	4	Lys173, His214, Arg211, and Gly203	Arg205, Ile215, and Arg204
Catechin	−6.0	1	5	Glu89, Leu44, Asp43, Lys139, and Ser140	Leu85, His214, Asp110, and Ala138
Cinnamic acid	−5.5	4	2	Thr145 and Gly162	Lys147 and Tyr160
Ferulic acid	−5.5	1	4	Lys95, Arg88, Phe78, and Thr77	Pro91, Glu75, Ile76, and Phe87
Gallic acid	−4.3	8	5	Tyr160, Gly162, Arg141, Gly144, and Thr145	Asp161 and Tyr143
Gentistic acid	−5.0	1	2	Arg372 and Ala371	Phe373, Tyr378, Arg379, Leu352, and Trp377
Quercetin	−7.2	1	5	Gln112, Glu89, Asp43, Leu44, and Lys139	Leu142, Leu85, Asp110, and Ala138
Rutin	−8.1	1	7	Leu44, Asp43, Ser140, Gln112, Asp110, His214, and Lys139	Gln218, Glu89, Leu85, Ala138, Leu142, and Phe46
Syringic acid	−4.0	9	3	Arg205, Ile206, and Lys173	Arg204, Ile215, Arg211, and Gly203
Vanillic acid	−5.3	1	2	Ser140 and Tyr80	Lys139, Gly111, and Ala138
5OE3					
Caffeic acid	−5.0	3	3	Tyr163, Asn84, and Phe208	Thr164, Ile95, Ala91, and Ser87
Catechin	−7.7	1	5	Gly279, Gly300, Thr164, Glu305, and Tyr378	Ile301, Gly302, Ala303, Asp382, Ser280, His394, and Ala278
Cinnamic acid	−5.7	4	2	Pro129 and Tyr25	Ala124, Ala125, Asn61, Ala108, Glu107, Arg128, Ser65, and Asp132
Ferulic acid	−6.4	1	2	Ser87 and Arg88	Asp94, Ala91, Ile83, Asn84, Ile95, and Pro171
Gallic acid	−5.9	3	5	Asp382, Tyr378, Glu305, Thr164, and Thr304	Ala303, Gly302, and Ile301
Gentisic acid	−5.7	3	4	Arg266, Pro263, Ala265, His294, and His293	Phe289 and Arg262
Quercetin	−8.4	1	6	Gly279, Gly300, Thr304, Glu305, Asp382, and His394	Pro281, Ala278, Gly302, Ile301, Ala303, Tyr378, Thr164, and Ser280
Rutin	−9.4	1	6	Gly300, Thr304, Glu305, Asp382, His394, Gly279	Ala278, Gly302, Ile301, Ala303, Tyr378, Thr164, and Ser280
Syringic acid	−6.2	2	4	Gly302, Asp382, Gly279, and Gly300	Ala278, Ile301, Pro281, His394, and Ser280
Vanillic acid	−5.9	3	4	Ala319, Ala391, Arg393, and Asp321	Tyr392, Glu3885, Arg386, and Asp387

**FIGURE 6:**
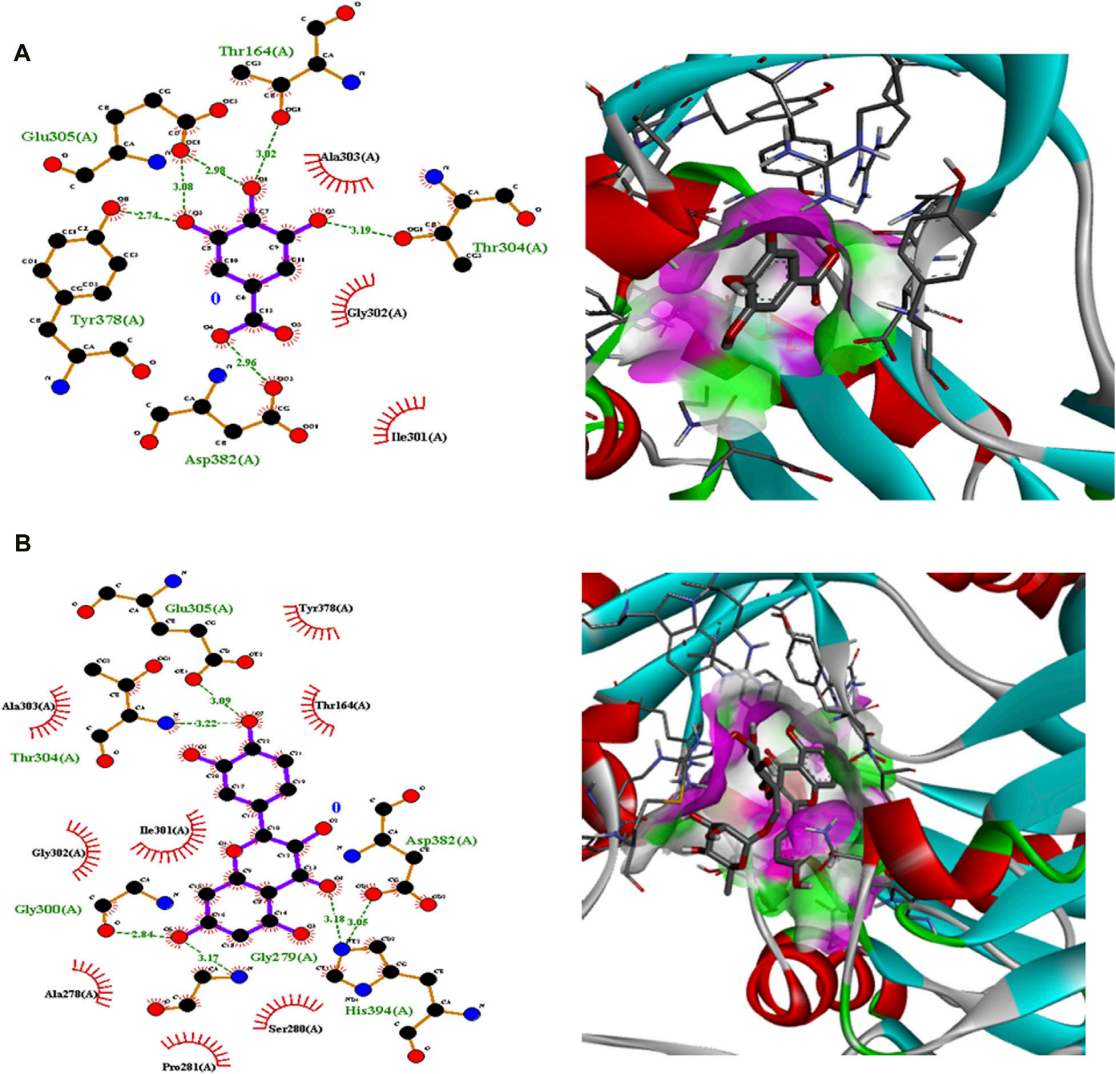
Interaction analysis of gallic acid pose 3 **(A)** and rutin pose 1 **(B)** with transcription regulator 5OE3.

### 3.4 Antimicrobial resistance pattern

Different antibiotics (14) were used to determine the antibacterial susceptibility of the isolated clinical oral bacterial strains, including *P. aeruginosa*, *B. chungangensis*, *B. paramycoides*, *and P. dendritiformis*. Almost all the isolated strains were resistant to antibiotics except imipenem and ciprofloxacin and, in a few cases, tetracycline (Table 6).

**TABLE 6:** Antibiogram studies of oral pathogens against selected antimicrobial agents.

Antibiotic	Bacterial strain zone of inhibition (mm)				
	*Bacillus chungangensis*-1	*Pseudomonas aeruginosa*	*Bacillus paramycoides*	*Bacillus chungangensis*-2	*Paenibacillus dendritiformis*
Amoxicillin	1	0	0	0	0
Amoxicillin clavulanic acid	0	0	0	0	3
Imipenem	15	11	17	10	16
Tetracycline	15	0	10	4	5
Ciprofloxacin	10	12	9	10	13
Piperacillin	10	3	1	0	15
Ceftriaxone	0	0	0	0	0
Cefoxitin	0	1	1	0	0
Meropenem	0	0	0	0	0
Polymyxin B	4	0	0	0	1
Sulfamethoxazole	0	0	0	5	5
Cefotaxime	0	0	0	0	0
Aztreonam	0	0	0	4	0
Gentamicin	0	4	6	4	6

Green, sensitive; red, resistant.

#### 3.4.1 Minimum inhibitory concentrations of different phenolic compounds against oral bacterial strains

The standard ATCC strains were investigated against polyphenolic compounds to compare the activity amongst resistant and standard strains. In the case of standard strains, all strains were susceptible to polyphenolic compounds. In the case of *B. paramycoides*, amongst clinical isolates, cinnamic acid, catechin, caffeic acid, and vanillic acid showed significant inhibition (MIC 0.25 mg/mL). Caffeic acid was highly active (MIC 0.25 mg/mL) against *P. aeruginosa*, while cinnamic acid showed inhibition against both strains of *B. chungangensis* (MIC 0.25 mg/mL) (Table 7). It was thus evident that polyphenolic compounds were active against resistant oral pathogens.

**TABLE 7:** Minimum inhibitory concentration of polyphenolic compounds against clinical strains.

S. no.	Compound name	MIC mg/mL				
		*Bacillus paramycoides*	*Pseudomonas aeruginosa*	*Bacillus chungangensis*	*Bacillus chungangensis*	*Paenibacillus dendritiformis*
1	Gentisic acid	0.5	0.25	0.25	0.25	0.125
2	Ferulic acid	0.5	0.5	0.5	0.25	0.0625
3	Gallic acid	0.5	0.5	0.5	0.25	0.125
4	Rutin	0.5	0.5	0.25	0.25	0.25
5	Cinnamic acid	0.25	0.25	0.125	0.25	0.25
6	Catechin	0.25	0.5	0.25	0.25	0.5
7	Quercetin	0.5	0.25	0.5	0.25	0.25
8	Caffeic acid	0.25	0.125	0.25	0.25	0.5
9	Syringic acid	0.5	0.5	0.25	0.25	0.5
10	Vanillic acid	0.25	0.5	0.5	0.25	0.25
11	Ciprofloxacin	0.031	0.0078	0.031	0.031	0.25

#### 3.4.2 Antibiofilm and antiquorum sensing activities

Among the 10 tested phenolic compounds, significant antibiofilm activity was recorded in the case of ferulic acid (60.3% ± 2.2%), catechin (73.5% ± 1.6%), quercetin (64.4% ± 1.4%), and vanillic acid (69.4% ± 1.3%) against *B. paramycoides* (Table 8). *P. aeruginosa* biofilm inhibition was reported by cinnamic acid (80.90% ± 1.1%) and caffeic acid (80.83% ± 1.6%), whereas quercetin presented significant inhibition (87.4% ± 1.4%) of *B. chungangensis-2*. Similarly, biofilms produced by *P. dendritiformis* were significantly inhibited by syringic acid (76.4% ± 1.2%) and vanillic acid (75.2% ± 1.6%) (Table 8). All the selected plant-origin phenolic compounds were checked for antiquorum sensing activity against *C. violaceum*, and no inhibition was noted (Supplementary Figure).

**TABLE 8:** Antibiofilm property of phenolic compounds against oral bacteria.

Compound	Concentration (mg/mL)	Percent biofilm inhibition of the biofilm produced by oral bacteria				
		*Bacillus paramycoides*	*Pseudomonas aeruginosa* *(%)*	*Bacillus chungangensis-1* *(%)*	*Bacillus chungangensis-2* *(%)*	*Paenibacillus dendritiformis* *(%)*
Gentisic acid	0.25	21.8% ± 1.1%	59.5 ± 0.12	11.4 ± 1.3	78 ± 2.1	35 ± 1.21
	0.125	3.7% ± 2.1%	29.5 ± 2.0	6.14 ± 1.21	73 ± 1.0	23.7 ± 1.4
Ferulic acid	0.25	60.3 ± 2.2	79.3 ± 1.2	7.4 ± 1.4	69 ± 0.42	21.2 ± 1.2
	0.125	22% ± 1.3%	71.4 ± 1.31	1.7 ± 0.2	15.6 ± 1.1	7.2 ± 1.4
Gallic acid	0.25	50% ± 1.4%	69.1 ± 2.4	18.8 ± 2.1	79 ± 1.5	58 ± 1.4
	0.125	34.6% ± 1.1%	65.2 ± 2.1	12.9 ± 1.4	58.6 ± 2.3	12.4 ± 1.2
Rutin	0.25	47.1% ± 2.1%	73.7 ± 0.45	23.35 ± 0.4	80 ± 1.8	45 ± 0.31
	0.125	40.4% ± 2.22%	53.99 ± 1.2	10.5 ± 0.6	21.8 ± 1.3	26.9 ± 0.45
Cinnamic acid	0.25	34.4% ± 1.2%	80.9 ± 1.1	31.4 ± 0.21	88 ± 1.21	59.5 ± 1.2
	0.125	29.9% ± 1.4%	62.4 ± 0.6	28.5 ± 0.23	23.8 ± 1.4	58.5 ± 1.6
Catechin	0.25	73.5% ± 1.6%	78.5 ± 0.81	20.8 ± 1.4	69.4 ± 0.48	62.7 ± 2.1
	0.125	53.7% ± 1.6%	44.67 ± 2.1	13 ± 1.3	34 ± 1.3	30.3 ± 2.4
Quercetin	0.25	64.4% ± 1.4%	71.4 ± 0.84	75 ± 2.0	87.4 ± 1.4	50.25 ± 1.4
	0.125	63.5% ± 2.1%	54.8 ± 1.6	52.3 ± 2.4	23.3 ± 1.6	25.5 ± 1.2
Caffeic acid	0.25	56.6% ± 1.4%	80.83 ± 1.6	33.8 ± 0.87	76.3 ± 0.85	81 ± 1.3
	0.125	28.2% ± 1.6%	77.2 ± 1.8	26.9 ± 2.7	7.6 ± 0.84	62 ± 1.4
Syringic acid	0.25	57.2% ± 1.3%	65.5 ± 2.1	17.6 ± 3.1	63.9 ± 2.1	76.4 ± 1.2
	0.125	48.1% ± 0.2%	42.7 ± 1.32	2.75 ± 1.6	27.8 ± 1.4	22.4 ± 1.2
Vanillic acid	0.25	69.4% ± 1.3%	72.4 ± 1.21	60.5 ± 1.7	34.2 ± 1.3	75.2 ± 1.6
	0.125	63.2% ± 1.6%	55.9 ± 1.4	25.6 ± 1.21	20.9 ± 1.4	19 ± 1.2
Ciprofloxacin	0.031	72.7% ± 2.1%	81 ± 1.31	54.4 ± 1.3	74.3 ± 1.2	52.6 ± 1.1
	0.015	67.2% ± 0.31%	65.7 ± 1.2	43.3 ± 1.2	56.18 ± 1.6	23.5 ± 0.47

## 4 Discussion

The oral cavity is a natural reservoir of diversified microbes, including *Staphylococcus* spp*.*, *Candida* spp*.*, *Granulicatella* spp., *Streptococcus* spp., *Veillonella* spp*.* (Gendron et al., 2000), and several transient bacteria (Singh et al., 2014). Most of these can colonize the oral cavity and develop biofilms that are resistant to commonly used antimicrobial agents (Rather et al., 2021). Considering emerging resistance in oral pathogens and limited treatment options, we investigated polyphenolic compounds for their antimicrobial and antibiofilm potential. Nevertheless, diabetic patients are more susceptible to oral bacterial infections, including periodontitis and gingivitis, and long-term complications of the cardiovascular system (Loesche, 1996). The bacterial strains were isolated from the dental plaque of female diabetic patients since, in Pakistan, female diabetic patients have high oral infection ratios due to poor hygiene. This investigation proposes a novel, safe, natural, and effective solution to these complicated cases. Initially, the drug likeness of polyphenolic compounds was assessed. All polyphenolic compounds followed drug rules, except rutin, which showed violations including a high molecular weight [>500], H-bond donors [>5], and H-bond acceptor [>10]. As stated in the literature, the violations must not be greater than 1; otherwise, the tested molecules may have limited oral absorption (Tijjani et al., 2022). This is based on the fact that Lipinski parameters indicate molecule bioavailability, lipophilicity, and permeation across cell membranes (Kumar et al., 2010; Kenny, 2022). In addition, the score function set by the Molsoft tool is also helpful in explaining the drug likeness of the molecule and the permissible range (−1 to +1) for a molecule to be considered a drug. It was observed that cinnamic acid (−1.17) showed a deviation from the standard value, i.e., < −1, and, therefore, was considered a violation. The oral bioavailability of the drug molecules was predicted using a bioavailability radar (SwissADME). The pink area on the radar is an indication of the bioavailability of the drug molecule, and various parameters of the drug must be within the range of the pink area for good bioavailability (Daina et al., 2017). Various parameters were taken into consideration, and it was observed that all tested molecules were within the colored area, indicating drug-like features except INSATU (instauration), which indicated slight unsaturation, slightly affecting the bioavailability of the tested polyphenolic compounds. The effects of the tested polyphenolic compounds on the CNS were predicted using the boiled egg model embedded in SwissADME, and it was evident that cinnamic acid, ferulic acid, and vanillic acid can permeate the brain, whereas all other tested molecules are only absorbed from the gastrointestinal tract. These findings propose a possible route for drug administration; however, the tested polyphenolic compounds were intended to produce a localized effect in the oral cavity and can be administered as an oral emulgel (Daina and Zoete, 2016). The ADMET analysis of drug molecules is an important computational estimation that enables the pharmacokinetic and pharmacokinetic attributes of molecules in drug repurposing and drug lead discovery (Guan et al., 2018). The ADMET analysis confirmed previous findings and further indicated that the tested molecules are neither substrates nor inhibitors of cytochrome P450 that reflect no modification in the liver. The cytochrome P450 family largely consists of over 50 enzymes. However, most of the drug molecules are metabolized by 2C19, D6, CYP1A2, 2E1, 2C92, 3A4, 3A4, and CYP2D6 (Sliwoski et al., 2013). Our findings suggested that most of the tested molecules were good drug candidates for oral intake, as reported previously (Sanz et al., 2015). The toxicity profile of the analyzed compounds indicated that all compounds were non-toxic to the skin except cinnamic acid. This is important since it ensures the usage of the analyzed compounds for the management of oral infections.

Molecular docking is an effective and extensively used technique to understand the molecular aspects of proteins and protein–ligand interactions in the drug discovery process (Murgueitio et al., 2012). The antimicrobial activity of flavonoids and polyphenols is certainly due to several mechanisms, specifically through direct damage to the outer envelope, genetic material, and interference in cell signaling (Donadio et al., 2021). In molecular docking investigations, the interaction analysis of polyphenolic compounds was performed against biofilm and quorum-sensing transcription regulators, including 1ROS, 4B2O and 5OE3. In the case of 1ROS, most H-bonding interactions were due to Asp17, Asp21, Glu12, Arg13, His7, Glu12, and His7 amino acids residing within active pockets. Furthermore, these interactions were stabilized through π–alkyl, π-stacked, and van der Waals interactions. Here, among flavonoids, the highest number (eight) of H-bonds was reported with rutin, involving Asp21, Arg13, Asp17, Glu12, Leu11, Lys14, His7, and Arg15 amino acids, and quercetin (five), with Thr144, Phe105, Ile107, Arg30, and Thr145 amino acids. Similarly, among phenolic acids, gallic acid presented the highest number of H-bond formations (six), involving Arg13, Asp17, Lys14, His7, Glu12, and Leu11 amino acids. In 4B2O, rutin presented the most stable complex formation with Leu44, Asp43, Ser140, Gln112, Asp110, His214, and Lys139 amino acids, which occurred due to strong H-bonding. Among phenolic acids, gallic acid showed the highest number of H-bond formations (five) with Tyr160, Gly162, Arg141, Gly144, and Thr145 amino acids. A nearly similar trend was recorded in the case of 5OE3 docking. Rutin showed highly stable complex formation with high free energy [−9.4 ΔG (kJ mol^‒1^)] involving Gly300, Thr304, Glu305, Asp382, His394, and Gly279. Among phenolic acids, gallic acid developed a stable H-bond formation with Asp382, Tyr378, Glu305, Thr164, and Thr304. This may indicate the potential inhibitory effect of the tested compounds on bacterial growth. The H-bond interactions are of great significance since they determine the stability and structure of proteins that are crucial in therapeutic effects (Bitencourt-Ferreira et al., 2019). The aromatic ring of phenolic compounds normally acts as a strong inhibitor owing to delocalized electrons on the aromatic rings and electron-donating and electron-withdrawing groups (Li et al., 2022). Similarly, the strong interaction of flavonoids is mainly attributed to specialized structural conformation (Abd Ghani et al., 2020). The phenolic acids are mainly provided with an aromatic ring supported by several phenolic and/or carboxylic groups at the ortho and meta positions. The specific position of these groups on the aromatic ring of phenolic acids is the main factor involved in strong H-bond formation (Oufensou et al., 2021). It was also noted that compounds with several phenolic groups have high free energy, as noted in the case of gallic acid in docking with all targets.

The clinical isolates were processed for antibiogram studies to investigate resistance levels against antibiotics. Ciprofloxacin, imipenem, and tetracyclines were active against clinical strains including *P. aeruginosa*, *Bacillus paramycoides, B. chungangensis-*1, *B. chungangensis-*2*,* and *P. dendritiformis,* whereas all other antibiotics showed resistance as mentioned in the CLSI criteria. Resistance to antimicrobial therapy has become alarmingly high, which has greatly increased the health burden (Kathia et al., 2020) in developing countries like Pakistan, where poverty, non-compliance, and lack of awareness represent the major contributing factors. Knowing the current status of antimicrobial resistance in an area can be an effective tool for designing newer strategies to cope with the situation (Parmanik et al., 2022). During our investigation, the isolated oral pathogens were resistant to most of the antimicrobial agents previously reported in an earlier investigation in Pakistan (Døving et al., 2020). This is quite alarming since, in addition to the development of severe infections in the oral cavity, such infections can lead to dental and oral–maxillofacial cavity disruption (Koren et al., 2011) and the development of several cardiovascular diseases (Meinen et al., 2021) due to long-term oral infections.

The MIC analysis of polyphenolic compounds was performed against clinical pathogens, and significant inhibition was recorded, ranging from 0.0625 to 0.25 mg/mL, which is interesting against resistant strains. The resistant strains are difficult to treat due to several reasons, including genetic mutations and biofilm formation (Dunai et al., 2019). Polyphenolic compounds are considered antibacterial since they can disrupt bacterial membranes, inhibit biofilm formation, and possess several virulence factors (Miklasińska-Majdanik et al., 2018). Furthermore, polyphenolic compounds are provided with strong antioxidant activity, and it has been established that these compounds lower oxidative stress by inhibiting the generation of reactive oxygen species (ROS), thereby inhibiting bacterial growth (Ispiryan et al., 2024). In our case, a concentration-dependent increase in activity was noted, which can be attributed to the increased inhibition of ROS. Our findings were consistent with those of previous reports that presented significant activity of polyphenolic compounds against resistant pathogens (Flemming et al., 2021).

The antibiofilm activities of polyphenolic compounds were performed against *B. paramycoides*, *P. aeruginosa*, and *P. dendritiformis*, and significant inhibition was observed against *B. chungangensis* and *P. dendritiformis*. In this investigation, polyphenolic compounds, including catechin, cinnamic acid, and quercetin, presented significant dose-dependent inhibition of isolated strains. Investigators have suggested that the antibiofilm inhibitory potential of polyphenolic compounds could be due to the downregulation of certain genes and a decrease in membrane permeability (Ivanov et al., 2022).

Cell–cell signaling or bacterial quorum sensing is an important mechanism involved in biofilm formation in addition to cell surface adhesion, an increase in membrane fluidity, and interference with energy mechanisms (Cushnie and Lamb, 2011). Polyphenolic compounds mainly have polar groups (more OH), which facilitate the easy entry of such molecules within exopolysaccharides (polymeric matrix) in the bacterial biofilm and affect cells (Pesci et al., 1999). In this investigation, no antiquorum sensing was recorded (zone of inhibition = 0 mm); it was therefore proposed that strong antibiofilm activity could possibly be due to other underlying mechanisms, including interference with membrane fluidity and energy mechanisms, cell wall, and DNA synthesis, as explained previously (Gafforov et al., 2024).

## 5 Conclusion

Plant polyphenolic compounds were investigated against resistant oral pathogens and their biofilms. Both the *in silico* and *in vitro* data suggested that polyphenolic compounds possess significant potential to eradicate the biofilm produced by resistant clinical oral bacteria. No antiquorum sensing activity was reported, which confirms that for biofilm formation, there can be another mechanism that can be explored in future investigations. Thus, the use of standardized plant extracts containing these polyphenolic compounds can be an interesting approach to designing herbal formulations for the oral cavity. The outcomes of the investigation are of great interest since they propose a natural treatment option for the management of oral bacterial biofilms. The pharmaceutical industry, especially the nutraceutical industry, can use the outcomes and proceed to use new nutraceutical polyphenolic formulations against oral bacteria. We further propose detailed *in vivo* and formulation design investigations.

## Data Availability

The GenBank accession number for the 16S rRNA gene sequence of strain Bacillus chungangensis (SUB14650934 2M_27F-725) is PQ147054, Pseudomonas aeruginosa (SUB14650934 Malp_27F-1075) is PQ147055, Bacillus paramycoides (SUB14650934 4M_27F-1050) is PQ147056, Bacillus chungangensis (SUB14650934 U5_27F-986) is PQ147057 and for strain Paenibacillus dendritiformis (SUB14650934 C14_27F-1050 ) is PQ147058.
