# Supplementary material for: Bioactive plant waste components targeting oral bacterial pathogens as a promising strategy for biofilm eradication
Source: Front Chem. 2024 Aug 9;12:1406869. doi: 10.3389/fchem.2024.1406869 (PMC11341444; doi:10.3389/fchem.2024.1406869)
Supplement: Supplementary file 1 [file DataSheet1.docx]

**Supplementary data**

**Resistant Oral Bacterial Biofilm Eradication by phenolic compounds using *in silico* and *in vitro* models.**


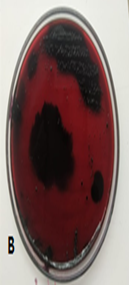

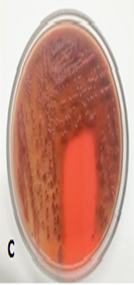

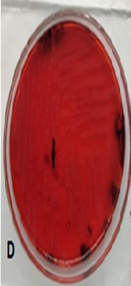

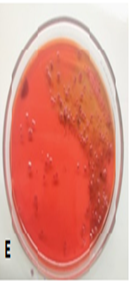

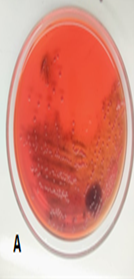


Figure S1.colonies of Bacillus chungangenesis (A), Pseudomonas aerogenosa (B), Bacillus chungangenesis (C), Paenibacillus dentiformis (D), Bacillus paramycoides (E) on congo red agar.


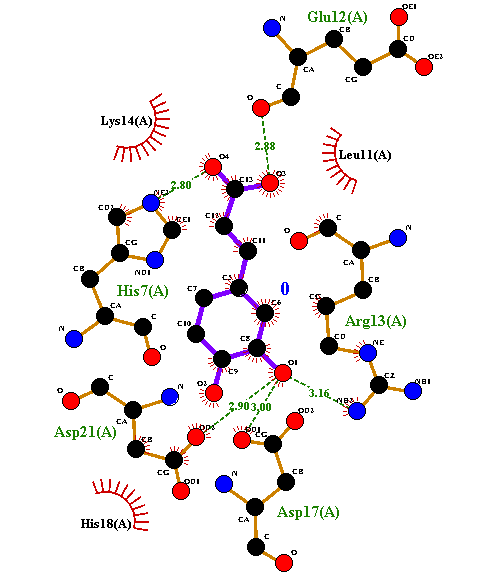

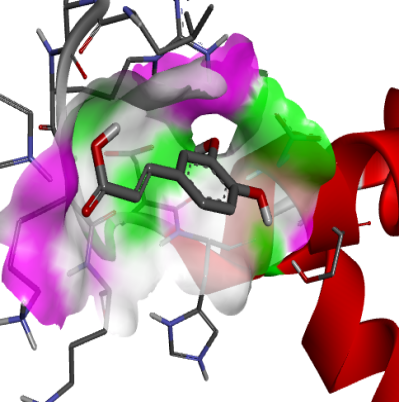


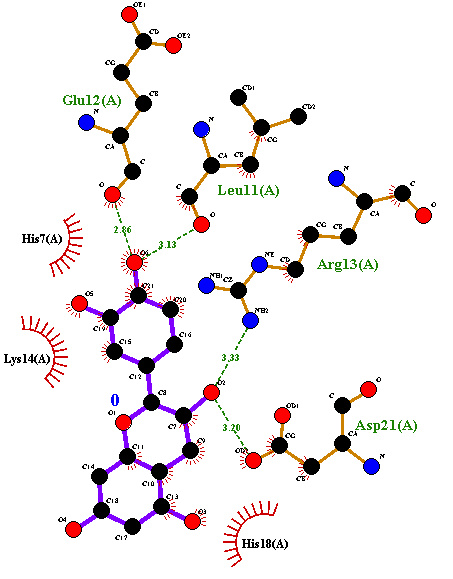
Figure S2 Interaction analysis of Caffeic acid pose 1 with transcription regulator IRO5.


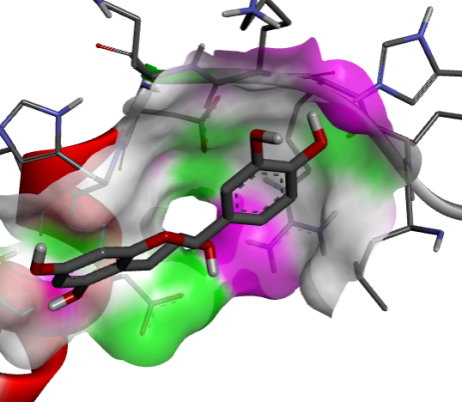


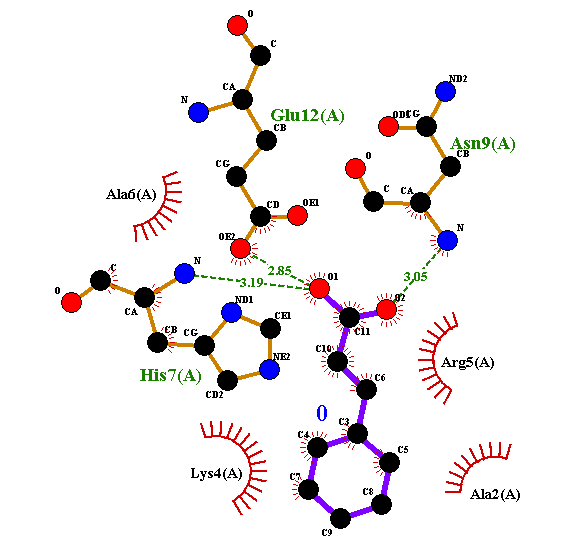
Figure S3 Interaction analysis of Catechin pose 3 with transcription regulator IRO5.


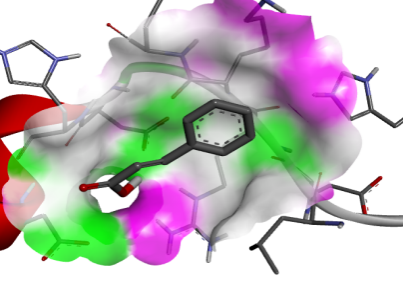


Figure S4. Interaction analysis of Cinnamic pose 5 with transcription regulator IRO5.


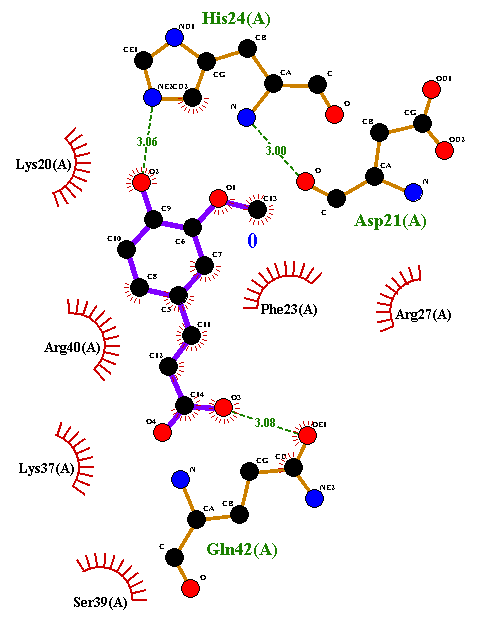

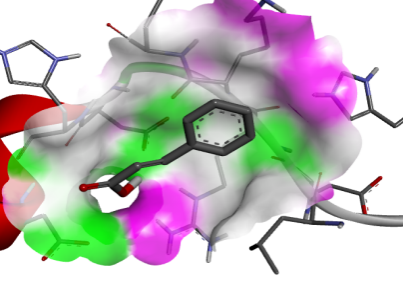


Figure S5. Interaction analysis of ferulic acid pose 1 with transcription regulator IRO5.


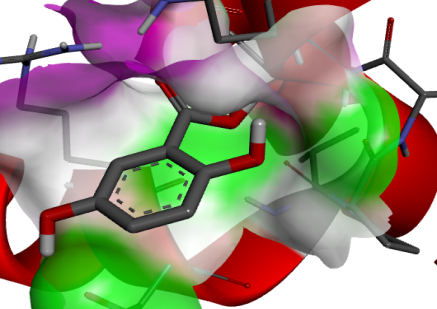

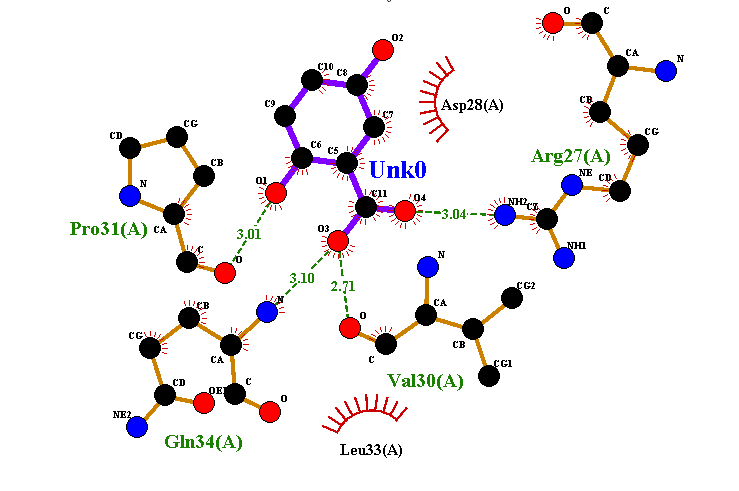


Figure S6 Interaction analysis of gentistic acid pose 1 with transcription regulator IRO5


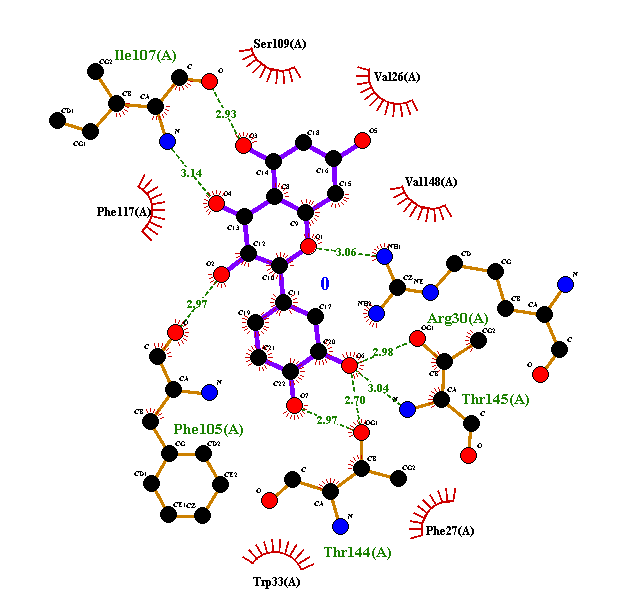

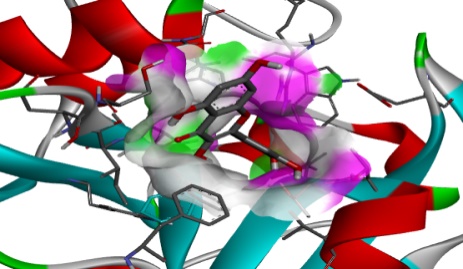


Figure S7. Interaction analysis of quercetin pose 1 with transcription regulator IRO5


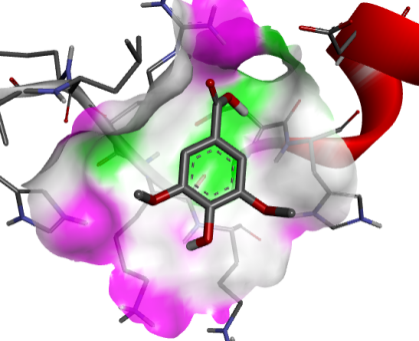

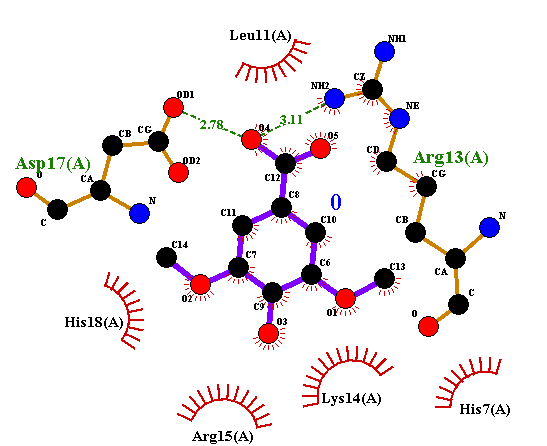


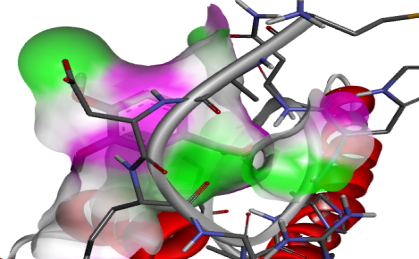

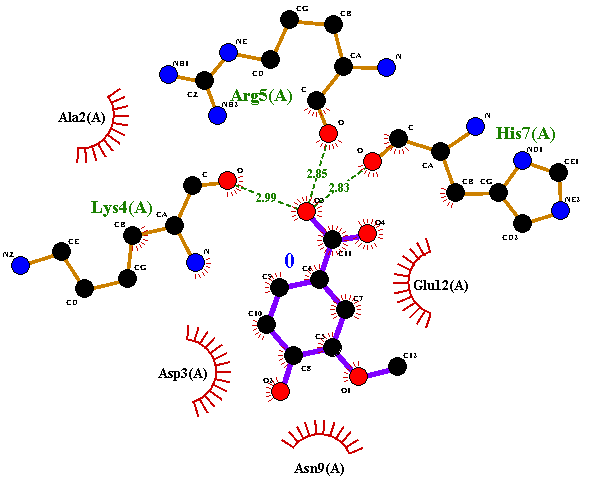
Figure S8. Interaction analysis of syringic acid pose 1 with transcription regulator IRO5.

Figure S9. Interaction analysis of vanillic acid pose 1 with transcription regulator IRO5.


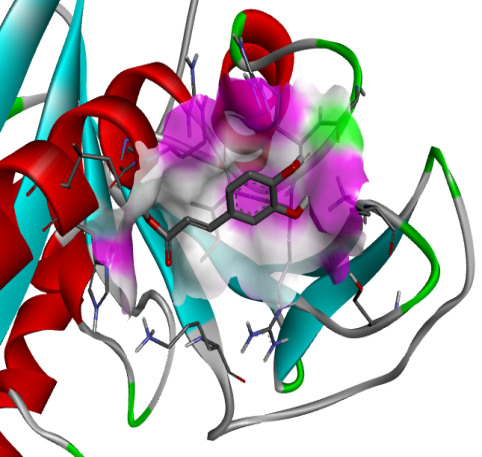


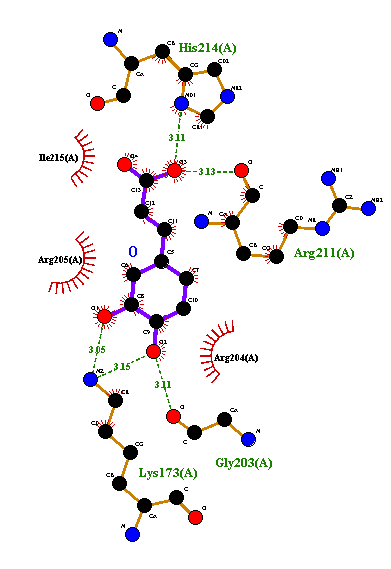


Figure 10. Interaction analysis of caffeic acid pose 5 with transcription regulator 4B2O


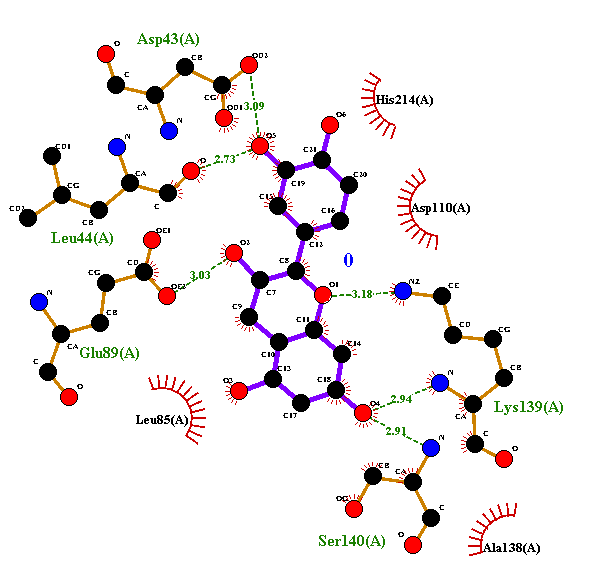

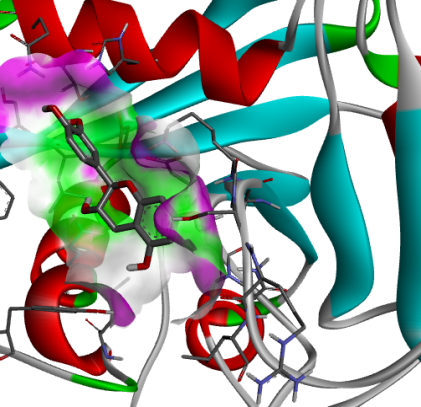


Figure S11. Interaction analysis of Catechin pose 1 with transcription regulator 4B2O


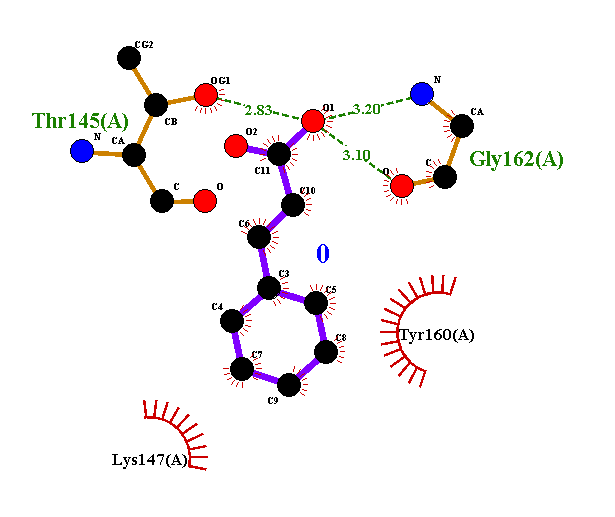


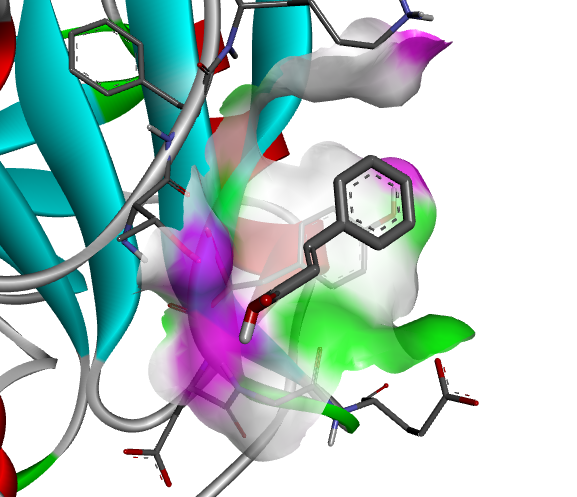


Figure S12. Interaction analysis of cinnamic acid pose 1 with transcription regulator 4B2O


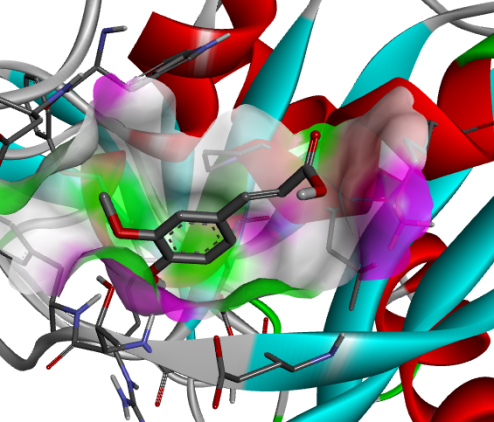

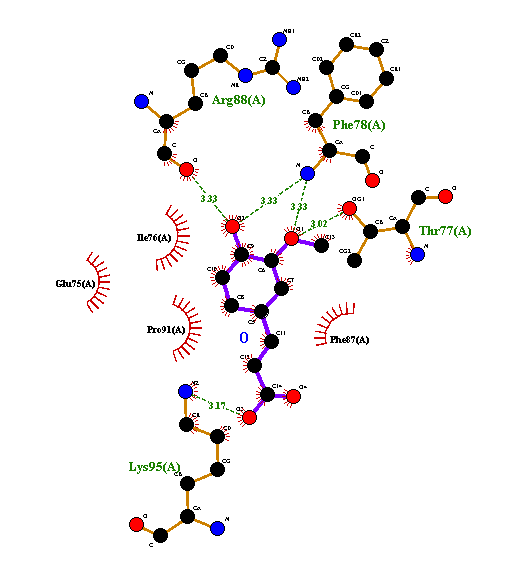


Figure S13. Interaction analysis of ferulic acid pose 1 with transcription regulator 4B2O


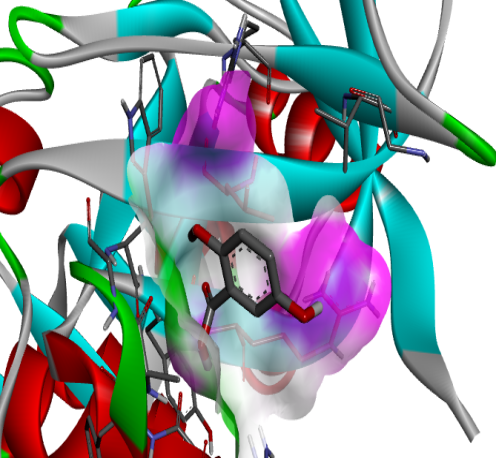

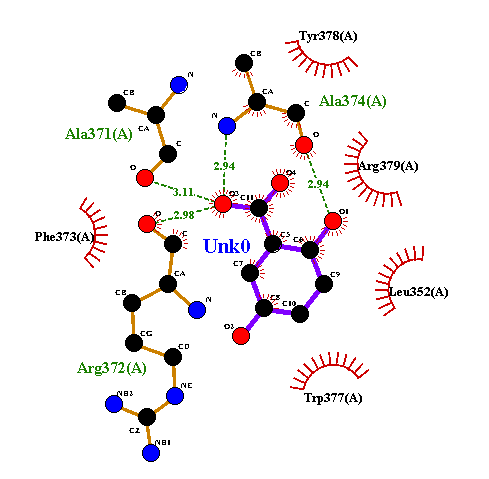


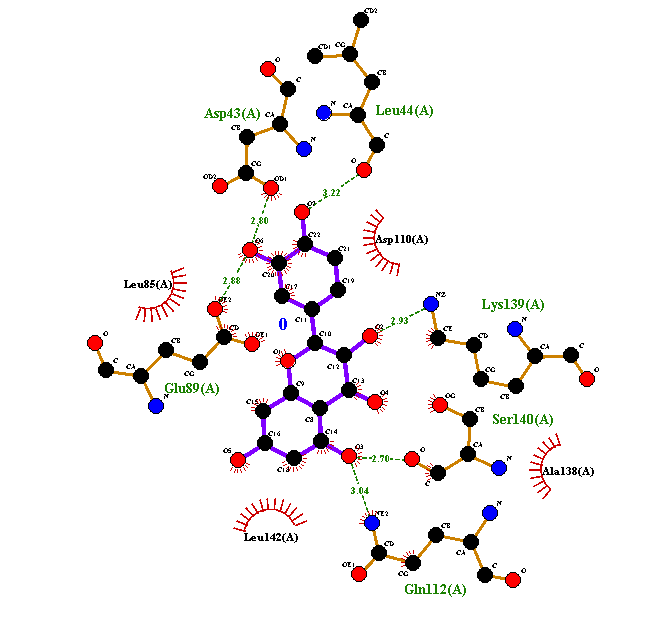

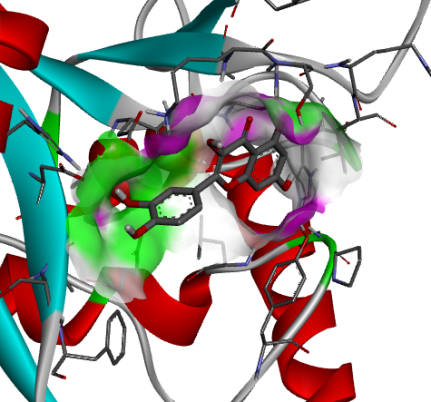
Figure S14. Interaction analysis of gentistic acid pose 1 with transcription regulator 4B2O

Figure 15. Interaction analysis of quercetin pose 1 with transcription regulator 4B2O


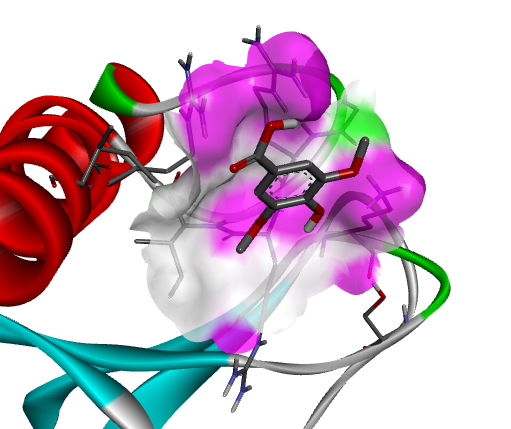

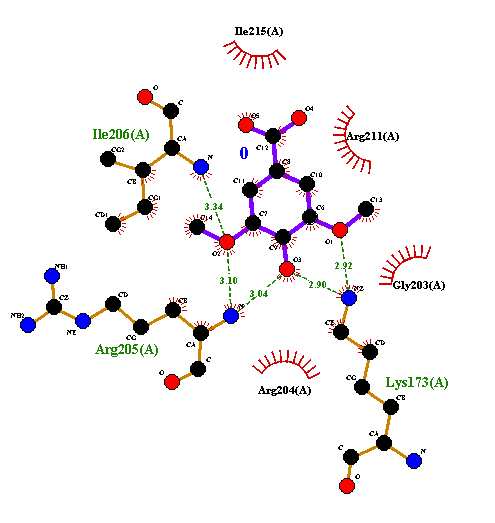


Figure 16. Interaction analysis of syringic acid pose 9 with transcription regulator 4B2O


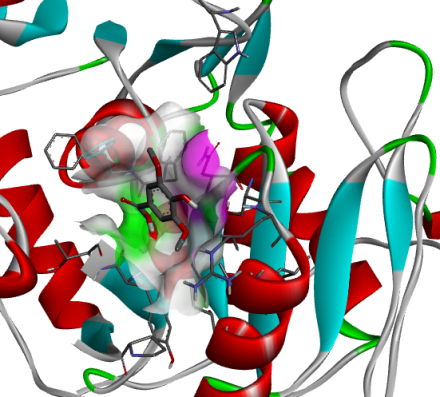

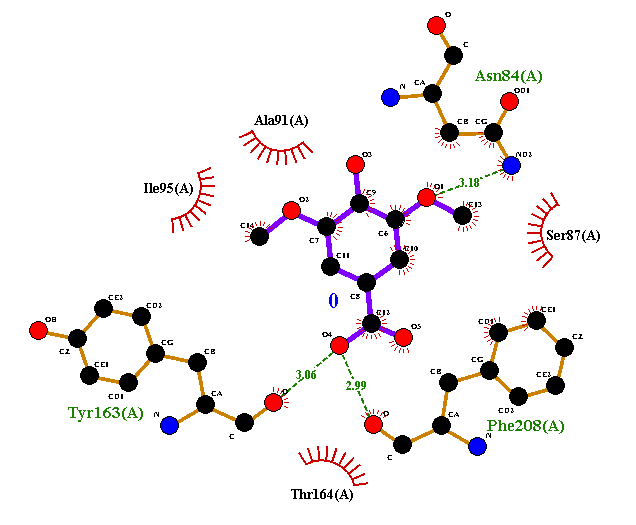


Figure 17. Interaction analysis of caffeic acid pose 3 with transcription regulator 5OE3


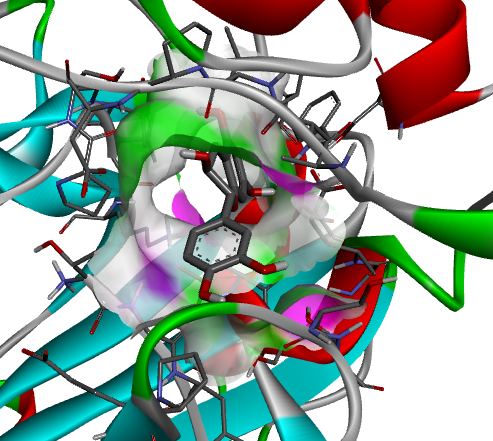

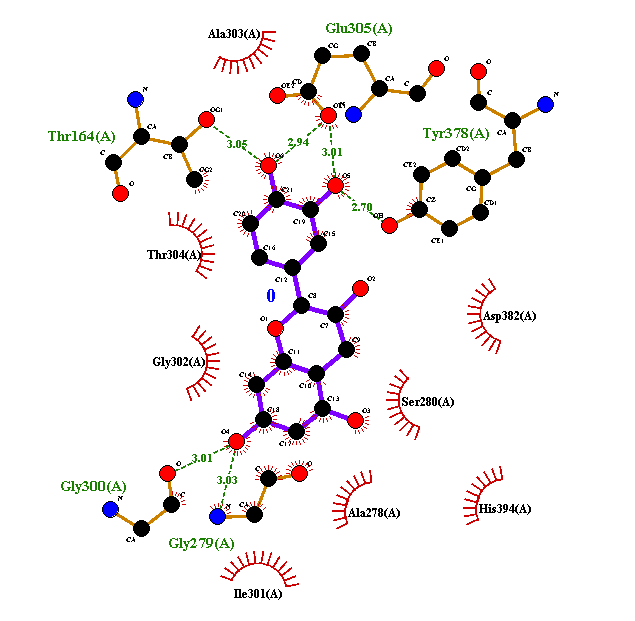


Figure S18 Interaction analysis of Catechin acid pose 1 with transcription regulator 5OE3


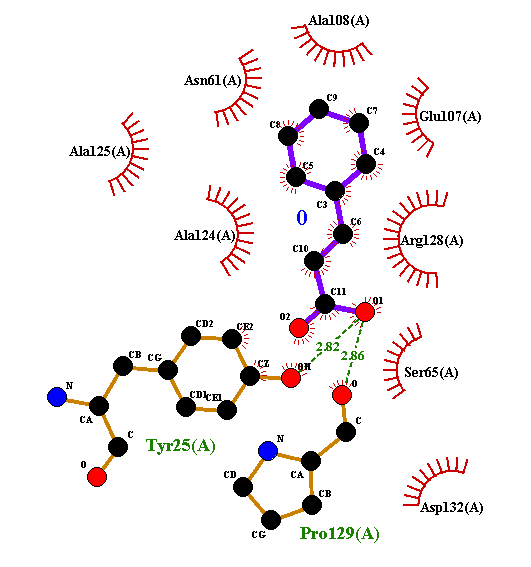

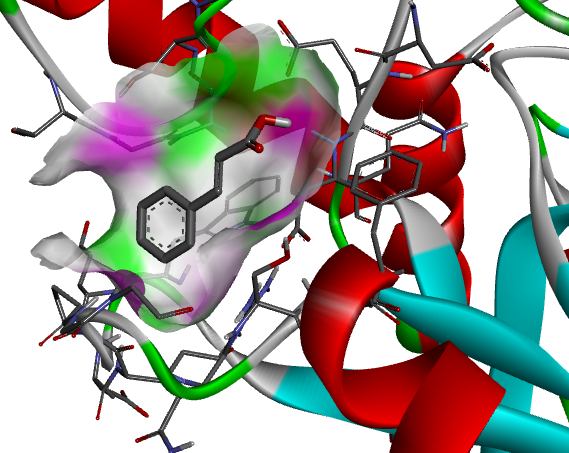


Figure S19. Interaction analysis of cinnamic acid pose 4 with transcription regulator 5OE3


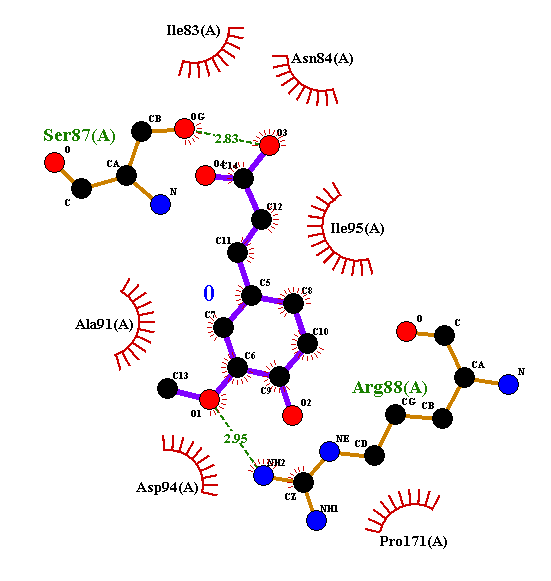

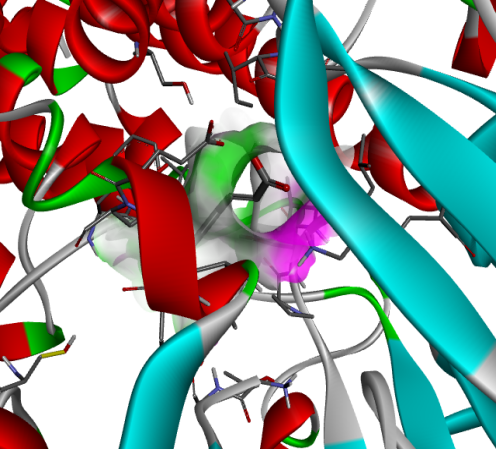


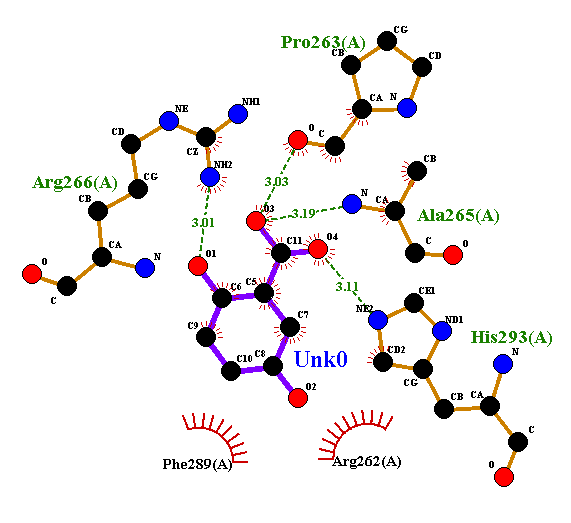
Figure 20. Interaction analysis of ferulic acid pose 1 with transcription regulator 5OE3


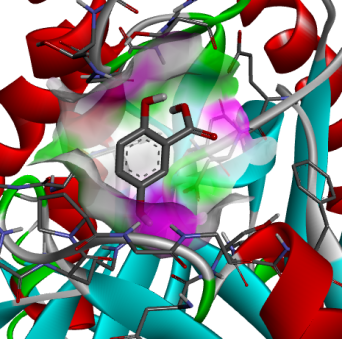


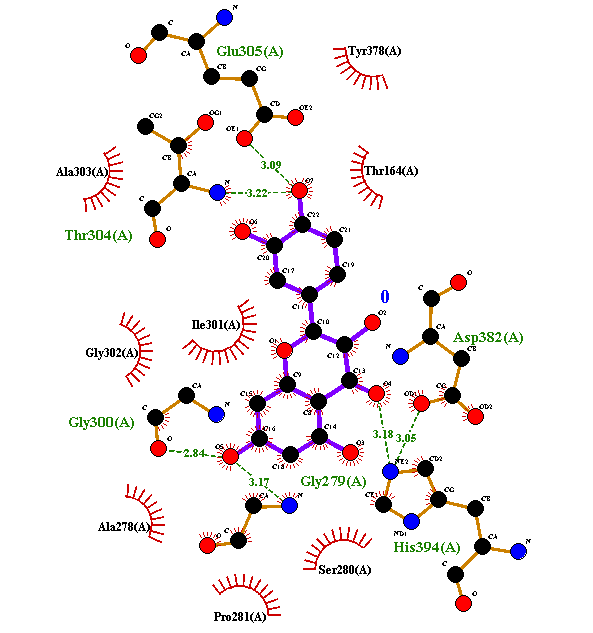
Figure 21. Interaction analysis of gentistic acid pose 3 with transcription regulator 5OE3


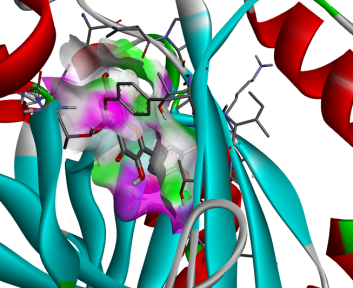


Figure 22. Interaction analysis of quercetin pose 1 with transcription regulator 5OE3


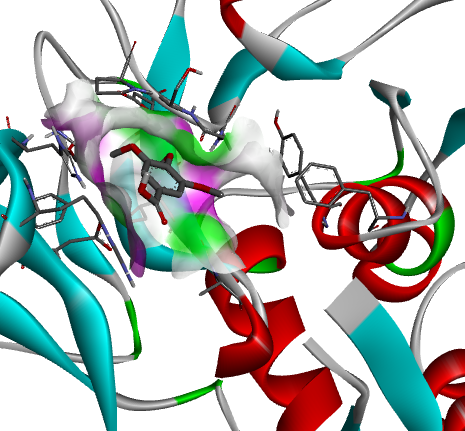

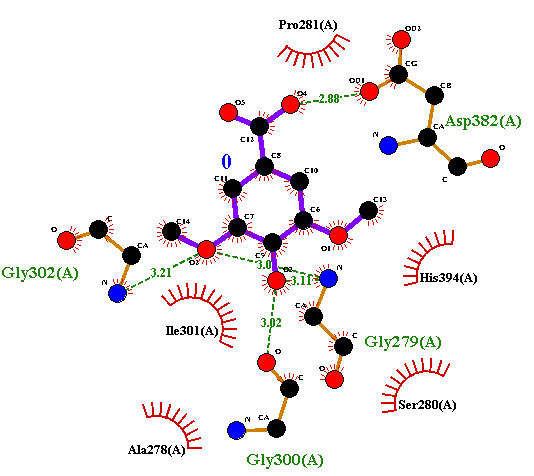


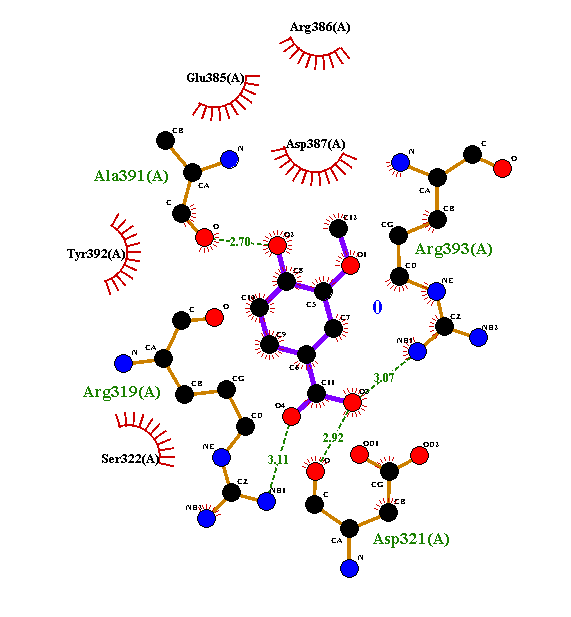
Figure S23. Interaction analysis of Syringic acid pose 2 with transcription regulator 5OE3


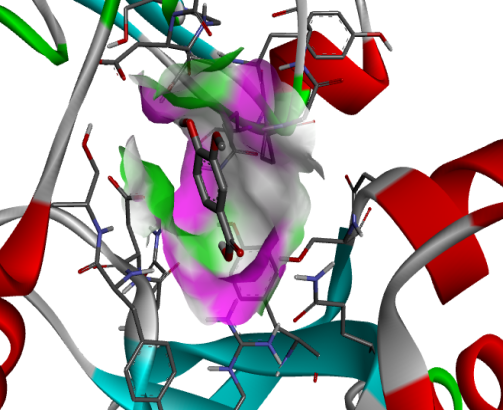


Figure S24 Interaction analysis of vanillic acid pose 3 with transcription regulator 5OE3


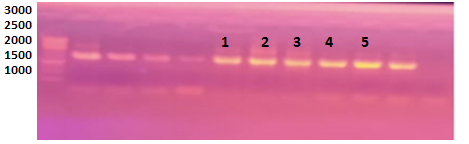


Figure S25. Gel electrophoresis of DNA strand obtained from individual bacterial strains.


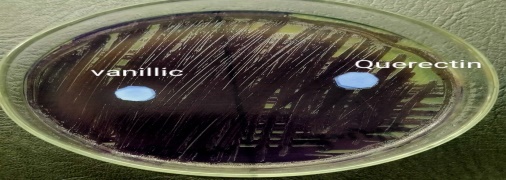

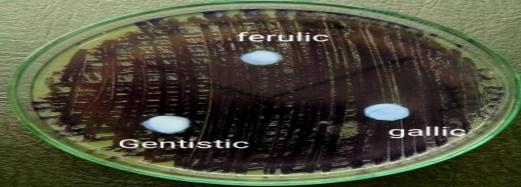

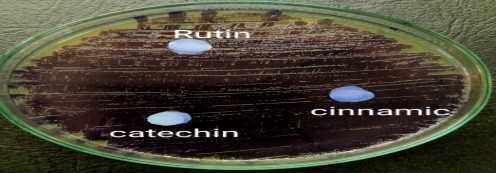

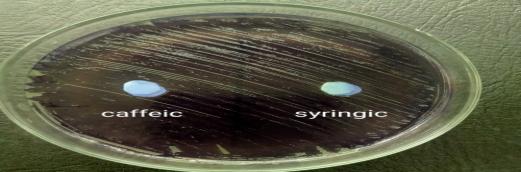


Figure S26. Antiqourum sensing activity of polyphenolic compounds

**Mathodology**

**DNA extraction**

The DNA was extracted through a method (Phenol/Chloroform method) with slight modification (Hai-Rong and Ning Jiang 2006). Here in this case the lysis step was skipped in which SDS/lysozyme or proteinase K and lysed the cells directly through phenol. First of all in order to extract the DNA from positive bacteria, 1 ml cell suspension was centrifuged at 8000g for 2 minutes. After the completion of the centrifugation the supernatant was removed, the isolated cells were then washed twice with STE buffer (100mM NaCl, 10 mM Tris/HCl, 1mM EDTA,pH 8.0). After that the isolated pallets were re-suspended in 200 micro liter TE buffer (10mM Tris/HCl, 1 mM EDTA, pH 8.0). Then 100 micro liter of the Tris-saturated phenol (pH 8.0) was added to theses followed by a vortex mixing step of 60s for bacteria to lyse cells. Then the samples were centrifuged at 13000g for 5 minutes to separate the organic phase from the aqueous phase. Then from the aqueous phase 160 micro liter was taken into a clean 1.5 ml tube. TE buffer 40 micro liter was added to make the volume 200 micro liter and mixed 100 micro liter chloroform and centrifuged again for the time duration of 5 minutes at the rate of 13000g at 4 °C. chloroform extraction was adopted to purify the lysate. The chloroform extraction procedure was repeated 3 times. Then the upper aqueous phase was transferred to the 1.5 ml clean tubes in the volume of 160 micro liter. In order to digest the RNA 40 micro liter and 5 micro liter of the RNase (10mg/ml) and stored at 37 °C for 10 minutes. Then to the tubes 100 micro liter of the chloroform was added and centrifuged at 13000g at 4 °C. The upper aqueouse phase (150 microliter) was then added to the 1.5 ml clean tube. Finally the aqueous phase composed of the purified DNA was stored at -20 °C and can be used directly as such.

**DNA quantification**

The quantity of DNA obtained was determined using the Qubit® 2.0 fluorometer and the Qubit® double straned DNA HS Assay kit (Life Technologies, Mulgrave, VIC, Australia), and the minimum amount that could be detected was 10 pg/l. To prevent any possible contamination, each PCR was conducted with and without cleaning the master mix. Four extraction controls (EC1-4) and two blank PCR controls (known as NT1 and NT2, which had no template) were employed. In order to eliminate any potential contamination, the master mix was decontaminated using the ArcticZymes® PCR Decontamination Kit, which contained a double-strand specific DNase and DTT. To achieve this, 0.5 microliter of dounle strand DNase and 0.5 microliter of DTT per 20 microliter reaction (adjusted for 50 endpoint reactions) were added to the master mix solutions, along with primers and probes for qPCR. The mixtures were then incubated at 37°C for 20 minutes for double strand DNase activation and 20 minutes at 60°C for double strand DNase inhibition in accordance with the manufacturer's guide lines. To prevent heat-related volume changes, the decontaminated master mix solutions were then cooled on ice for two minutes. (Stinson et al., 2018)

**16S rRNA gene qPCR**

The bacterial DNA levels in dsDNase-treated and crude extractions and PCR controls were compared using real-time PCR. The specific primers 891F (5'-TGGAGCATGTGGTTTAATTCGA-3') and 1033R (5'-TGCGGGACTTAACCCAACA-3') were used to amplify the V6 region of the 16S rRNA gene, as represented earlierly by Stinson et al. in 2018. The reaction mixture consisted of a 20 µl volume, which included 5 µl of either blank extraction control or nuclease-free water from Integrated DNA Technologies, Inc. (Queenstown, Singapore), 1X TaqMan Fast Advanced Master Mix from Applied Biosystems (Foster City, CA), 0.9×10-6 mol l-1 of each forward and reverse primers, 0.25×10-6 moll-1 of probe (5'-FAM-CAC-GAGCTGACGACARCCATGCA-TAMRA-3'), and 4.2 µl of water. The ViiA 7 Real-Time PCR System using Taqman Fast settings was used to the sample and for the amplication 40 cycles were performed. It was noted that All the loaded samples were duplicated (Stinson et al., 2018).

**16S rRNA gene sequencing**

The Agencourt AMPure XP Reagent was used to purify the PCR products in according to the guide lines of the manufacturer. The purified amplicons were suspended in Low TE buffer, and the Ion Plus Fragment Library Kit was used to create the NGS library as per the manufacturer's guidelines. End Repair Enzyme Mix was used to blunt-end the already purified PCR products (amplicon). After that at the same time while doing nick repairing the amplicon was attached with Ion Xpress Barcodes as well as Ion P1 adaopter. Agencourt AMPure XP reagent was used to purify the batteries. After the completion of the purification process these were amplified by using the platinum PCR ToughMix High Fidelity and Library Amplification. Specific thermal cycling conditions were adapted to mix the primer. The provided thermal conditions included denaturation for 5 minutes at 95°C. Then it was followed by 5 cycles of denaturation at 95°C and 58°C for fifteen seconds respectively and then at 70°C for the time period of only one minute. At this stage Qubit double strand DNA HS assay kit was used for measuring after the cleaning of the libraries properly using the reagent addressed previously in this experiment. The range of each library was adjusted to 100-1012 mol l-l in the Low TE Buffer to guarantee the even representation of each barcoded library in the sequencing reaction. The untreated samples were added in equal parts (13:1) to the final pool in equimolar concentration. Templates were prepared automatically by using the Ion Chwf System with Ion 520 and 530 Ext Kit and load chips using isothermal amplification technology from Thermo Fisher Scientific. Initially for templating onto ISPs and loadng on an ion 530 Chip a 50 microliter of the pooled library sample with the concentration of 100 10-12 mol 1-1 was loaded onto the ion S5 Ext Reagent cartridge. Finally, the Ion S5 ExT Sequencing Kit on an Ion S5 Sequencer with Torrent Suite Software ver. 5.2.2 using Default Calibration from Thermo Fisher Scientific was used to sequence the loaded hip for 1300 flows (Stinson et al., 2018).

Table S1 Sequencing data of ***Pseudomonas aeruginosa*** obtained after successful Sequencing

| Strain Code | Similarity | Sequence |
| --- | --- | --- |
| Malep | *Pseudomonas aeruginosa* | TCAATTTACTTCATTGTAGTATACGCATGCAGTCGAGCGGATGAATGGAG  CTTGCTCCTGGATTCAGCGGCGGACGGGTGAGTAATGCCTAGGAATCTGC  CTGGTAGTGGGGGATAACGTCCGGAAACGGGCGCTAATACCGCATACGTC  CTGAGGGAGAAAGTGGGGGATCTTCGGACCTCACGCTATCAGATGAGCCT  AGGTCGGATTAGCTAGTTGGTGGGGTAAAGGCCTACCAAGGCGACCATCC  ATAACTGGTCTGAGAGGATGATCAGTCACACTGGAACTGACACACGGTCC  AGACTCCTACGGGAGGCAGCAGTGGGGAATATTGGACAATGGGCGAAAGC  CTGATCCAGCCATGCCGCGTGTGTGAAGAAGGTCTTCGGATTGTAAAGCA  CTTTAAGTTGGGAGGAAGGGCAGTAAGTTAATACCTTGCTGTTTTGACGT  TACCAACAGAATAAGCACCGGCTAACTTCGTGCCAGCAGCCGCGGTAATA  CGAAGGGTGCAAGCGTTAATCGGAATTACTGGGCGTAAAGCGCGCGTAGG  TGGTTCAGCAAGTTGGATGTGAAATCCCCGGGCTCAACCTGGGAACTGCA  TCCAAAACTACTGAGCTAGAGTACGGTAGAGGGTGGTGGAATTTCCTGTG  TAGCGGTGAAATGCGTAGATATAGGAAGGAACACCAGTGGCGAAGGCGAC  CACCTGGACTGATACTGACACTGAGGTGCGAAAGCGTGCGGAGCAAACAG  GATTAGATACCCTGGTAGTCCACGCCGTAGACGATGTCGACTAGCCGTTG  GGATCCTTGAGATCTTAGTGGCGCAGCTAACGCGATAAGTCGACCGCCTG  GGGAGTACGGCCGCAAGGTTAAAACTCACATGAATTGACGGGGGCCCGCA  CAAGCGGTGGAGCATGTGGTTTAATTCGAAGCAACGCGAAGAACCTTACC  TGGCCTTGACATGCTGAGAACTTTCCAGAGATGGATTGGTGCCTTCGGGA  ACTCAGACACAGGTGCTGCATGGCTGTCGTCAGCTCGTGTCGTGAGATGT  TGGGTTAAGTCCCGTAACGAGCGCAACCCTTGTCCTTAGTTACCAGCACC  TCGGGGGGGCACTCTAAGGAGACTGCCGGTGACAAACCCGAAGAAAAGTG  TGGGATGAAATTCAAGTCATCATGGGCCCTTTCTGGCGGGGCTCCCACCT  GGTTCCATGGTGCGGTGTCAAAGGGTGTCCCACCCCGGGGGGGGAACTCA  TCCCCTAAAAAACCAACTATCTCCGGATCTCCTTCTGCCCCCCCCCCCCT  GAATCTCCCACCCCTTTTATATCCTGAAAAAAAAATGTCCGGTGAAAACT  TTCTCCGCCGCCTTTT |


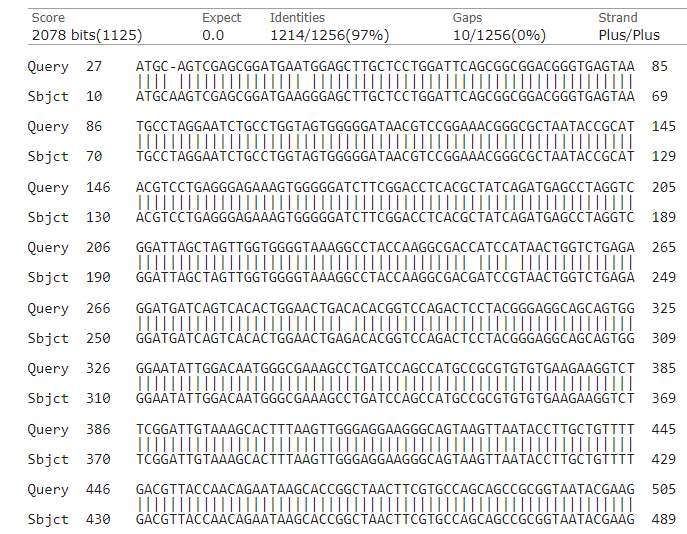


Figure S27. *Pseudomonas aeruginosa* BLAST sequence alignment results

Table S2 Sequencing data of ***Bacillus chungangensis*** obtained after successful Sequencing

| Strain Code | Similarity | Sequence |
| --- | --- | --- |
| 2M | *Bacillus chungangensis* | TGAGGGGCGGCGGCTACACATGCAAGTCGAGCGAACCGAAGAGAGCTTGC  TCTCTGAAGTTAGCGGCGGACGGGTGAGTAACACGTGGGCCACCTGCCTG  TAAGACTGGGATAACTTGCGGAAACGTGAGCTAATACCGGATACGCTTTT  ATATTCTCCTGAATATGAAAGGAAAGATGGCTTTTGCTATCACTTACAGA  TGGGCCCGCGGCGCATTAGCTAGTTGGTGAGGTAACGGCTCACCAAGGCG  ACGATGCGTAGCCGACCTGAGAGGGTGATCGGCCACACTGGGACTGAGAC  ACGGCCCAGACTCCTACGGGAGGCAGCAGTAGGGAATCTTCCGCAATGGA  CGCAAGTCTGACGGAGCAACGCCGCGTGAGTGAAGAAGGTTTTCGGATCG  TAAAACTCTGTTGTTAGGGAAGAACCCGTACCGTTCAAATAGGGCGGTAC  GCTGACGGTACCTAACCAGAAAGCCACGGCTAACTACGTGCCAGCAGCCG  CGGTAATACGTAGGTGGCAAGCGTTGTCCGGAATTATTGGGCGTAAAGCG  CGCGCAGGCGGTTTCTTAAGTCTGATGTGAAAGCCCACGGCTCAACCGTG  GAGGGTCATTGGAAACTGGGAGACTTGAGTGCAGAAGAGGAGAGTGGAAT  TCCACGTGTAGCGGTGAAATGCGTAGAGATGTGGAGGAACACCAGTGGCG  AAGGCGGCTCTCTGGTCTGTAACTGACGCTGAGGCGCGAAAGCGTGGGGA  GCGAACCGGATTAGATACCCTGGTAGTCCACGCCGTACACCATTAGTGCT  AACTGTTAAGGGGGTTTCCGCCCCCTTAGTGCTCTAGCGTACACATTTTG  CACAACCCCCCCGGGAGTATGGCCCCCGCGTTTTAACTCTAAAGAAGTAA  ACAACGGGCGCCCCGCAACATGTGGGAGCATGTTGGTTGTTTTAAATAAA  CAGCACCACGAAATATATCAACGGGGGTGGTGGTACTTCTATTATTATTC  TGTTGTTTTGTTTGGTGGTCCCCCTCCGGGGGGAACCAAAAGGGGGGTAG  GTGGGGGTTTTCCCCCCCCAATATGTGGTTTTTATTTATTTATGAAAAAC  CCCCCCCCCCCCCCGCCCCCCCCCCTCCTCTCCTTTTTTATGTCGCCCCC  CTCCTGTGGGGGGGGGAGAACCTCCCCCCCCCCCCCCCCCCCCCCAAAAA  AAAAAAAAGGGGGGGGAGGGGGTGGGGTTTAATACCTCTTTGTATTACAC  CCCCCCCTCCCCCTCCAGGGGGGGGGGCCACCCCCCCCCTGCCCCTGGGG  GGGGGGTTACCTCCTCGCGCACACGATGTT |


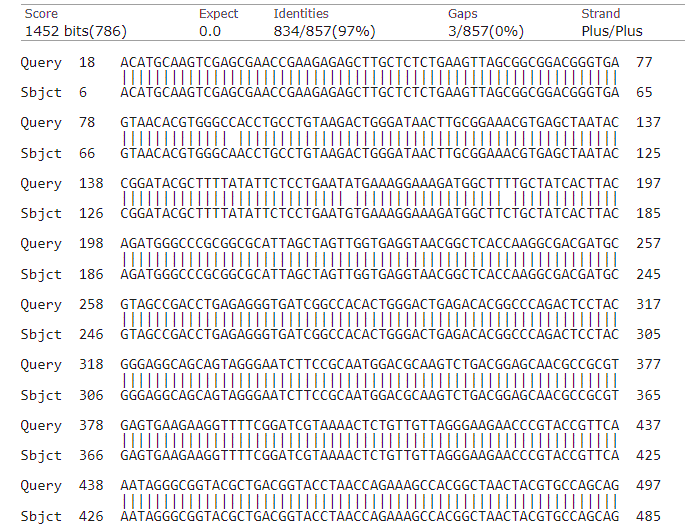


Figure: *Bacillus chungangensis* BLAST sequence alignment results

Figure S28. *Bacillus chungangensis* BLAST sequence alignment resultsTable S3 Sequencing data of *Bacillus paramycoides* obtained after successful Sequencing

| Strain Code | Similarity | Sequence |
| --- | --- | --- |
| 4M | *Bacillus paramycoides* | TCCCGTTGGGGGGTGCCTATACTGCAGTCGAGCGAATGGATTAAGAGCTT  GCTCTTATGAAGTTAGCGGCGGACGGGTGAGTAACACGTGGGTAACCTGC  CCATAAGACTGGGATAACTCCGGGAAACCGGGGCTAATACCGGATAACAT  TTTGAACCGCAGGGTTCGAAATTGAAAGGCGGCTTCGGCTGTCACTTATG  GATGGACCCGCGTCGCATTAGCTAGTTGGTGAGGTAACGGCTCACCAAGG  CAACGATGCGTAGCCCACCTGAGAGGGTGATCGGCCACACTGGGACTGAG  ACACGGCCCAGACTCCTACGGGAGGCAGCAGTAGGGAATCTTCCGCAATG  GACGAAAGTCTGACGGAGCAACGCCGCGTGAGTGATGAAGGCTTTCGGGT  CGTAAAACTCTGTTGTTAGGGAAGAACAAGTGCTAGTTGAATAAGCTGGC  ACCTTGACGGTACCTAACCAGAAAGCCACGGCTAACTACGTGCCAGCAGC  CGCGGTAATACGTAGGTGGCAAGCGTTATCCGGAATTATTGGGCGTAAAG  CGCGCGCAGGTGGTTTCTTAAGTCTGATGTGAAAGCCCACGGCTCAACCG  TGGAGGGTCATTGGAAACTGGGAGACTTGAGTGCAGAAGAGGAAAGTGGA  ATTCCATGTGTAGCGGTGAAATGCGTAGAGATATGGAGGAACACCAGTGG  CGAAGGCGACTTTCTGGTCTGTAACTGACACTGAGGCGCGAAAGCGTGGG  GAGCAAACAGGATTAGATACCCTGGTAGTCCACGCCGTAAACGATGAGTG  CTAAGTGTTAGAGGGTTTCCGCCCTTTAGTGCTGAAGTTAACGCATTAAG  CACTCCGCCTGGGGAGTACGGCCGCAAGGCTGAAACTCAAAGGAATTGAC  GGGGGCCCGCACAAGCGGTGGAGCATGTGGTTTAATTCGAAGCAACGCGA  AGAACCTTACCAGGTCTTGACATCCTCTGACAACCCTAGAGATAGGGCTT  CTCCTTCGGGAGCAGAATGACAGGTGGTGCAGGGTTGTCGTCACCTCCTG  TCCTGAGAATTTTGGGTTAAATCCCCCAACCAAGGGCAACCCTTGAACTT  AATTGCCACCATTTTATTTGGGCACTCCTAAGGGGACTGCCGGTGACAAA  CCCGAAGAAAGGGGGGGGGAGAAACCTCCAAAAACACCAGCCGCCCTTTT  TAAACGGGGGGGCACACCCTTGTTAAAATGGGGAGGGTACAAAGAAGGTG  CCCAAAACCCGCGGGGGGGGGAGGGAATCTCCAAAAAACCTTTTTTTTTT  TTTGGAATTGGGGGGCCCACCCCC |


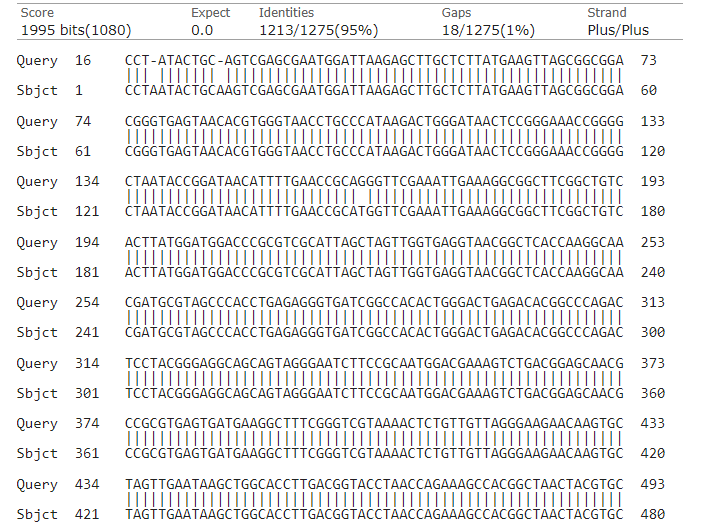


Figure S29. *Bacillus paramycoides* BLAST sequence alignment results

Table S4.Sequencing data of *Bacillus chungangensis* obtained after successful Sequencing

| Strain Code | Similarity | Sequence |
| --- | --- | --- |
| *U5* | *Bacillus chungangensis* | CCGCATTAAATGCGTGCCTATACATGCAAGTCGAGCGAACCGAAGAGAGCTTGCTCTCTGAAGTTAGCGGCGGACGGGTGAGTAACACGTGGGCAACCTGCCTGTAAGACTGGGATAACTTGCGGAAACGTGAGCTAATACCGGATACGCTTTTATATTCTCCTGAATGTGAAAGGAAAGATGGCTTTTGCTATCACTTACAGATGGGCCCGCGGCGCATTAGCTAGTTGGTGAGGTAACGGCTCACCAAGGCGACGATGCGTAGCCGACCTGAGAGGGTGATCGGCCACACTGGGACTGAGACACGGCCCAGACTCCTACGGGAGGCAGCAGTAGGGAATCTTCCGCAATGGACGCAAGTCTGACGGAGCAACGCCGCGTGAGTGAAGAAGGTTTTCGGATCGTAAAACTCTGTTGTTAGGGAAGAACCCGTACCGTTCAAATAGGGCGGTGCGCTGACGGTACCTAACCAGAAAGCCACGGCTAACTACGTGCCAGCAGCCGCGGTAATACGTAGGTGGCAAGCGTTGTCCGGAATTATTGGGCGTAAAGCGCGCGCAGGCGGTTTCTTAAGTCTGATGTGAAAGCCCACGGCTCAACCGTGGAGGGTCATTGGAAACTGGGAGACTTGAGTGCAGAAGAGGAGAGTGGAATTCCACGTGTAGCGGTGAAATGCGTAGAGATGTGGAGGAACACCAGTGGCGAAGGCGGCTCTCTGGTCTGTAACTGACGCTGAGGCGCGAAAGCGTGGGGAGCGAACAGGATTAGATACCCTGGTAGTCCACGCCGTAAACGATGAGTGCTAAGTGTTAGGGGGTTTCCGCCCCTTAGTGCTGCAGCAAACGCATTAAGCACTCCGCCTGGGGAGTACGGCCGCAAGGCTGAAACTCAAAGGAATTGACGGGGGCCCGCACAAGCGGTGGAGCATGTGGTTTAATTCGAAGCAACGCGAAGAACCTTACCAGGTCTTGACATCCTTCGCTACTTCTAGAGATAGAAGGTTCCCCTTCGGGGGACGAAATGACAGGTGGTGCATGGTTGTCGTCACCTCGTGTCGTGAGATGTTTGGGTTAAGTCCCGCACCAACGCCACCCTTAAGCTTATTTGCCTCCTTTAATTGGGGCCTCCTAAATGTACTGCCGGTGACAACCGGAGGAAGGGGGGGGGTGAGGTCAATCTTCCTGCGCCCTTTGAACCGGGGGTACCACCTGCTTCCATGGGTGTGTCAACGGGGAGAACACCCGCGGGGGGACCATCCCCAAAAAACCTTTTTTTTTTTTTTATGTTTGGGGGCGCCTCCCCCCCCCTTTAAACCCCAATATCTTTTATTTCCG |


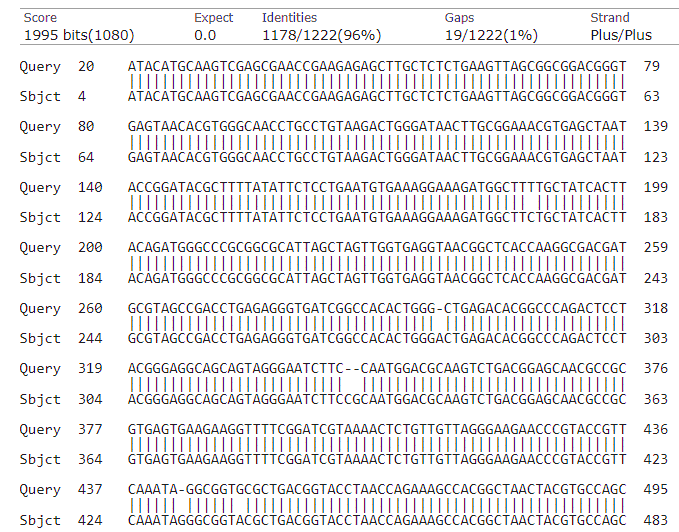


Figure S30. ***Bacillus chungangensis*** BLAST sequence alignment results.

Table S5. Sequencing data of ***Paenibacillus dendritiformis*** obtained after successful Sequencing

| Strain Code | Similarity | Sequence |
| --- | --- | --- |
| *C14* | *Paenibacillus dendritiformis* | CCGCATTAAATGCGTGCCTATACATGCAAGTCGAGCGAACCGAAGAGAGC  TTGCTCTCTGAAGTTAGCGGCGGACGGGTGAGTAACACGTGGGCAACCTG  CCTGTAAGACTGGGATAACTTGCGGAAACGTGAGCTAATACCGGATACGC  TTTTATATTCTCCTGAATGTGAAAGGAAAGATGGCTTTTGCTATCACTTA  CAGATGGGCCCGCGGCGCATTAGCTAGTTGGTGAGGTAACGGCTCACCAA  GGCGACGATGCGTAGCCGACCTGAGAGGGTGATCGGCCACACTGGGACTG  AGACACGGCCCAGACTCCTACGGGAGGCAGCAGTAGGGAATCTTCCGCAA  TGGACGCAAGTCTGACGGAGCAACGCCGCGTGAGTGAAGAAGGTTTTCGG  ATCGTAAAACTCTGTTGTTAGGGAAGAACCCGTACCGTTCAAATAGGGCG  GTGCGCTGACGGTACCTAACCAGAAAGCCACGGCTAACTACGTGCCAGCA  GCCGCGGTAATACGTAGGTGGCAAGCGTTGTCCGGAATTATTGGGCGTAA  AGCGCGCGCAGGCGGTTTCTTAAGTCTGATGTGAAAGCCCACGGCTCAAC  CGTGGAGGGTCATTGGAAACTGGGAGACTTGAGTGCAGAAGAGGAGAGTG  GAATTCCACGTGTAGCGGTGAAATGCGTAGAGATGTGGAGGAACACCAGT  GGCGAAGGCGGCTCTCTGGTCTGTAACTGACGCTGAGGCGCGAAAGCGTG  GGGAGCGAACAGGATTAGATACCCTGGTAGTCCACGCCGTAAACGATGAG  TGCTAAGTGTTAGGGGGTTTCCGCCCCTTAGTGCTGCAGCAAACGCATTA  AGCACTCCGCCTGGGGAGTACGGCCGCAAGGCTGAAACTCAAAGGAATTG  ACGGGGGCCCGCACAAGCGGTGGAGCATGTGGTTTAATTCGAAGCAACGC  GAAGAACCTTACCAGGTCTTGACATCCTTCGCTACTTCTAGAGATAGAAG  GTTCCCCTTCGGGGGACGAAATGACAGGTGGTGCATGGTTGTCGTCACCT  CGTGTCGTGAGATGTTTGGGTTAAGTCCCGCACCAACGCCACCCTTAAGC  TTATTTGCCTCCTTTAATTGGGGCCTCCTAAATGTACTGCCGGTGACAAC  CGGAGGAAGGGGGGGGGTGAGGTCAATCTTCCTGCGCCCTTTGAACCGGG  GGTACCACCTGCTTCCATGGGTGTGTCAACGGGGAGAACACCCGCGGGGG  GACCATCCCCAAAAAACCTTTTTTTTTTTTTTATGTTTGGGGGCGCCTCC  CCCCCCCTTTAAACCCCAATATCTTTTATTTCCG |


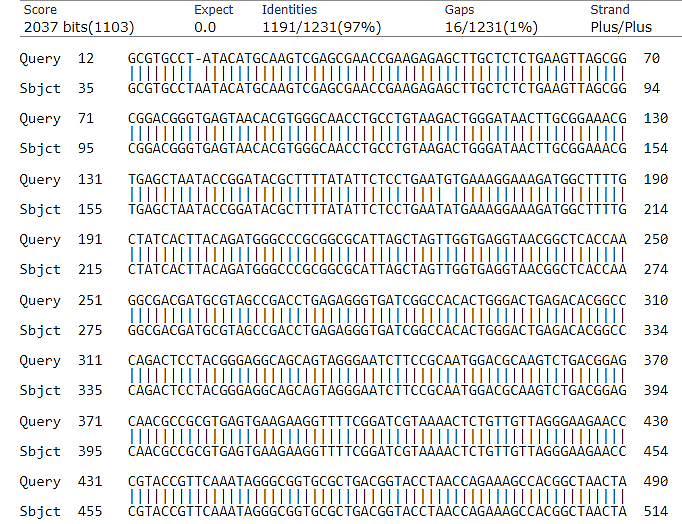


Figure S30. *Paenibacillus dendritiformis* BLAST sequence alignment results
